# Simvastatin suppresses spinal cord metastasis of medulloblastoma at clinically significant doses

**DOI:** 10.1038/s41419-025-07829-0

**Published:** 2025-07-15

**Authors:** Charley Comer, Kian Cotton, Christopher Edwards, Xiaoyang Dai, Sara Badodi, Roberto Buccafusca, Chris Bennett, Andrew Peet, Alice Williams, David Michod, Elena Bochukova, Maria Victoria Niklison-Chirou

**Affiliations:** 1 Life Science Department, University of Bath, Bath, UK; 2 Blizard Institute, Barts and The London School of Medicine and Dentistry, Queen Mary University of London, London, UK; 3 School of Physical and Chemical Sciences, Queen Mary University of London, London, UK; 4 Institute of Cancer and Genomic Sciences, University of Birmingham, Birmingham, UK; 5 Cancer Section, Development Biology and Cancer Programme, UCL Great Ormond Street Institute of Child Health, London, UK; 6 Centre for Therapeutic Innovation, Life Science Department, University of Bath, Bath, UK

**Keywords:** Metastasis, CNS cancer

## Abstract

Medulloblastomas (MBs) are aggressive brain cancers and represent the most common primary malignant tumour in children. Current treatment protocols involve an intensive regimen of surgery, radiation therapy and chemotherapy, guided by histopathology and risk stratification. Unfortunately, disease relapse proves fatal in 30% of cases, and treatment efficacy is compromised as MB cells develop resistance. Therefore, there is a critical need for more effective and tolerable therapies, especially for the treatment of aggressive MBs associated with a poor prognosis. Lipid metabolism reprogramming, characterized by increased cholesterol synthesis, lipid uptake and the activation of de novo lipogenesis, is a newly identified hallmark of cancers. Cholesterol is an essential structural component of membranes that contributes to membrane integrity and fluidity. Recently, increasing evidence has indicated that cholesterol is a major determinant by modulating cell signalling events governing the hallmarks of cancer. Our research demonstrates there is an overexpression of cholesterol metabolism in group 3 (G3), and group 4 (G4) MB subgroups compared to Sonic Hedgehog (SHH)-MB subgroup. In these tumours, cholesterol metabolism supports cell migration through the Rho-GTPase signalling pathway. Notably, we observed that shifting the culture conditions from 2D to 3D significantly upregulates lipid metabolism. Furthermore, spheroids derived from G3/G4-MBs and SHH-MBs show similar sensitivity to low doses of simvastatin. We validated these findings in a xenograft mouse model, where treatment with low doses of simvastatin led to increased survival time and remarkably, also reduced the metastatic spread of MB cells to the spinal cord. These results suggest that simvastatin holds potential as an adjuvant treatment for patients with medulloblastoma.

## Introduction

Medulloblastomas (MBs) are the most common malignant paediatric brain tumour, accounting for ~20% of cases [1]. These are embryonal tumours which develop in the cerebellum [2], and their diagnosis is based on clinical symptoms, imaging, histopathological and molecular analysis. MBs primarily occur in children and adolescents, with 70% of cases affecting children <16 years of age and a peak incidence at age 7 [3].

MBs are a heterogeneous group of tumours which have been divided by transcriptional profiling into four subgroups named wingless (WNT), sonic hedgehog (SHH), group 3 (G3) and group 4 (G4). Each have distinct demographics, molecular aberrations and clinical outcomes [4–6]. In 2016, these subgroups were incorporated into the WHO’s classification of central nervous system (CNS) tumours [7] marking a shift toward biologically informed diagnosis. However, substantial heterogeneity exists within each subgroup. For example, in SHH medulloblastomas, TP53 mutations are associated with significantly worse outcomes [2, 3] and amplification of MYC or MYCN is used for risk stratification, particularly in patients aged 3 years or older [8–10]. These molecular features are now critical to prognosis and treatment planning.

Improved characterisation and treatment of MBs has led to a huge increase in patient survival, with a current 70% survival rate at 5 years from diagnosis, risen from 30% in the 1960s [11, 12]. However, this figure has remained stagnant in recent decades. MBs-related fatalities account for a disproportionately high number of childhood cancer deaths, comprising ~10% [12]. This highlights an urgent need for the development of novel therapeutic approaches for the treatment of MBs, particularly for aggressive subtypes with poorer patient outcomes.

Reprogramming of lipid metabolism is a newly recognised hallmark of cancer, where tumour cells enhance de novo lipogenesis and lipid uptake to increase intracellular lipid storage that can be catabolised to generate energy in nutrient-deprived environments [13, 14]. The mevalonate pathway (MVA) is an important metabolic pathway which regulates the biosynthesis of cholesterol, an important component of cell membranes and cell signalling. Intermediates of the MVA pathway such as farnesyl pyrophosphate and geranylgeranyl pyrophosphate are required for post-translational modification of proteins involved in cellular growth and differentiation, a process known as prenylation, where proteins are farnesylated or geranylgeranylated [15, 16]. Examples of prenylated proteins include Ras and Rho-GTPases [17], both of which are involved in various cellular mechanisms from cell proliferation to cell motility, cell cycle progression from G0/G1 phase and angiogenesis [18, 19]. Their involvement in these cellular processes is centred around the formation of actin microtubules and remodelling of the actin cytoskeleton, thought to be the main process driving cancer cell migration [20]. Recently, Ras- and Rho-GTPases have been identified as essential for the metastasis of gastric and oesophageal cancers [21, 22].

The MVA can be inhibited by the use of statins, which competitively and irreversibly bind to 3-hydroxy-3-methylglutaryl coenzyme-A (HMG-CoA), and thus inhibit the activity of HMG-CoA reductase (HMGCR), the rate-limiting enzyme of the mevalonate pathway [23]. Statins are widely used for the treatment of hypercholesterolaemia and cardiovascular diseases and hold a very good safety profile [24]. There are several statins available, two of which are lipophilic with the ability to cross the blood-brain-barrier: lovastatin and simvastatin [16, 25]. Previous studies have shown the ability of high doses of statins to induce apoptosis in different brain tumours such as glioblastoma and MBs through decreased activation of the anti-apoptotic protein Bcl-2 and induction of cell cycle arrest in bladder cancer cells [26, 27].

Several studies have focused on the importance of the MVA in breast, gastric and bone cancer metastasis, however very little of its importance in paediatric brain tumour metastasis has been explored. This study aims to evaluate the effect of clinically significant doses of simvastatin on MBs cell migration and invasion.

## Materials and methods

### Cell culture

Medulloblastoma cell lines (DAOY, UW228-2, D425, D458 and ICb-1299) were cultured using methods previously described by Merve et al. [28]. DAOY and UW228-2 cells belong to the SHH-MB subgroup while D425, D458 and ICb-1299 are models of G3 and G4-MB subgroups. Briefly, MB cells were cultured in Dulbecco’s Modified Eagle’s Medium (DMEM; Gibco^TM^, US) with of GlutaMAX ^TM^, supplemented with 1% MEM Non-Essential Amino Acids Solution, 10% foetal bovine serum (FBS) and 1% penicillin-streptomycin-glutamine (Gibco™, US). Cells were incubated at 37 °C and 5% CO_2_ and sub-cultured every 2–3 days. DAOY, UW228-2 and ICb-1299 cells were grown as an adhesive monolayer, while D425 and D458 cells were grown as semi-adherent cultures.

MB cell lines DAOY and UW228-2 were obtained from ATCC; D425 and D458 cell lines were obtained from Dr Silvia Marino, Queen Mary University of London, and ICb-1299 cells were obtained from Dr Xiao-Nan Li, Baylor College of Medicine, Texas Children’s Cancer Centre, USA.

### Simvastatin preparation

Simvastatin powder (20 mg) purchased from US Pharmacopeia (USP, US) was reconstituted in 2.5 mL pure EtOH with 750μL NaOH and heated at 50 °C for 2 h to activate the prodrug. The pH was adjusted to 7 with 1 M HCl and then made up to 5 mL with EtOH to make a stock concentration of 1.1945mM.

### Immunofluorescence (IF)

MB cells were cultured on coverslips coated with Poly-L-Ornithine (PLO) and laminin (R&D Biosystems, US) followed by fixation with 4% PFA as described [28]. Coverslips were then washed 3 times with phosphate buffered saline (PBS), permeabilised at room temperature for 10 min using 0.3% Triton-X (Sigma, US), washed 3 times with PBS and then blocked for 1 h at room temperature using 5% goat serum in 0.1% PBS-T. Coverslips were then incubated at 4 °C for 48 h with anti-HMGCR (#ab174830, Abcam, UK), anti-FDPS (#PA5-28228, Invitrogen, US), anti-SREBP2 (#PA1-338, Thermo Fisher) and anti-farnesyl (341286, Calbiochem, Germany) primary antibodies at 1:50 dilution. After incubation with AlexaFluor 594 anti-rabbit secondary antibody and DAPI (1:5000), coverslips were mounted onto glass slides using Fluoromount-G^TM^ (Invitrogen, US). Five random fluorescent micrographs were taken using the EVOS M5000 microscope 20× objective, where fluorescence intensity was determined using ImageJ software, and values expressed as mean ± SD. All experiments were carried out in triplicate.

### Western blotting

Total protein was extracted from cell pellets using ice-cold RIPA buffer (25 mM Tris-HCl, pH 7.6, 150 mM NaCl, 1% Nonidet P-40, 1% sodium deoxycholate, 0.1% SDS with PhosSTOP^TM^ and protease inhibitor cocktail), as previously described [28]. Samples were vortexed for 30 s, left on ice for 30 min to lyse cells and then sonicated in an ice bath for 15 min to minimise genomic DNA contamination. Samples were then centrifuged at 4 °C for 15 min at 12,000 rpm to pellet cell debris, and supernatant transferred into a new Eppendorf tube. Samples were stored at −20 °C in aliquots until required to minimise freeze-thawing.

Protein separation was carried out using SDS-PAGE (4–20% Mini-PROTEAN® TGX™ Precast Protein Gels, 10-well, 30 µl, Bio-Rad, US), followed by transfer to 0.45 μm nitrocellulose membrane (Amersham/GE Healthcare, US) at 70 V for 1 h on ice. Membrane was blocked with 5% milk or BSA in TBST buffer (25 mM Tris-HCl, 137 mM NaCl and 0.1% Tween-20, pH 7.5) for 1 h at room temperature, followed by incubation with primary antibodies anti-RhoA/Cdc42/Rac1/phospho-Rac1 (Rho-GTPase Antibody Sampler Kit #9968, Cell Signalling Technology, US), anti-SREBP2 (#PA1-338, ThermoFisher, US) and vinculin (#sc-59803, Santa Cruz Biotechnology, US) at 1:1000 dilutions in 5% milk, and 2% BSA for phospho-Rac1. Blots were imaged using the Li-COR Odyssey® CLx imaging system (LI-COR, US).

### Cell viability

Cell viability assays were carried out using CellTiter-Glo® Luminescent Cell Viability Assay (Promega, US), following the manufacturer’s protocol. Cells were seeded at 5000/well (DAOY, D425, D458 and ICb-1299) and 10,000 cells/well (UW228-2) in 96-well plates (opaque-bottom, white; Nunc, Denmark) in triplicate. After 24 h, simvastatin was added at increasing concentrations from 0 to 50 µM. Viability of cells was measured after 48 h of simvastatin treatment with a CLARIOstar plate reader and Mars analysis software (BMG LABTECH, UK).

### In vitro transwell migration and invasion assays

These assays were performed as per published protocols listed in Pijuan et al. [29] (2019, DOI: doi.org/10.3389/fcell.2019.00107). For migration assays, Sarstedt 24-well plate transwell inserts (pore size 8 µm) were seeded with 5000 cells/well in low serum medium. Chemoattractant of complete medium (10% FBS) was added to the bottom of the chamber and cells left to migrate (24 h for adherent cell lines DAOY, UW228-2 and ICb-1299, 48 h for suspension cell lines D425 and D458). After incubation, cells were fixed with 4% PFA and washed with PBS. Non-migrated cells were cleaned from the upper membrane, and migrated cells on the bottom part of the membrane stained using 1% crystal violet. Membranes were then imaged using the EVOS M5000 microscope and average number of migrated cells calculated using ImageJ software, where values were expressed as mean ± SD. All experiments were conducted in triplicate and statistical analysis carried out using GraphPad Prism software.

### MTT assay

MTT (3-[4,5-dimethylthiazol-2-yl]-2,5 diphenyl tetrazolium bromide, Sigma, US) assays were also performed to assess cell viability. Cells were seeded to the same densities in clear-bottomed 96-well plates (Greiner AG, Austria) and treated with increasing concentrations of simvastatin from 0 to 100 µM. After 48 h, media was removed and replaced with MTT solution pre-diluted in complete DMEM to a final concentration of 0.45 mg/mL and incubated for 2–4 h. Once formazan crystals were present, media was removed and crystals dissolved using DMSO before absorbance was read with a Tecan Infinite 200 microplate reader (Tecan, Switzerland).

### Annexin V/PI

DAOY cells were seeded in 12-well plates. After 24 h, the medium was replaced by a fresh medium, and simvastatin (5 or 10 µM) was added. After treatment for 48 h, floating and adherent cells were collected. After centrifugation, the supernatant was removed, and cells were resuspended in 400 µL of Becton Dickinson (BD) Annexin V Binding Buffer (Cat. No.556454). Follow, cells were stained with 2 µl Annexin V-FITC (BD, Cat.No. 556420) and incubate at RT for 15 min. PI (5 mg/ml) was added. 20,000 events were collected.

### Wound closure assay

This assay was performed as per published protocols previously listed [29]. DAOY and UW228-2 cells were plated in tissue culture-treated 12-well plates (Nunc, Denmark) until they reached confluence. Cells were then incubated for 24 h with simvastatin (2 µM and 5 µM) before a wound was incited using 200 µL pipette tip down the centre of the well. After the wound was made, cell monolayers were washed two times with PBS to remove excess floating cells and debris, and media replaced with 1% FBS DMEM. Cells were then incubated and imaged at three different positions across each scratch were imaged at 0 h and after 21 h using the EVOS M5000 Live Imaging microscope (ThermoFisher Scientific, US). Gap closure analysed using ImageJ software. Statistical analysis of percentage gap closure was carried out with GraphPad Prism software using one-way ANOVA with multiple comparisons.

### Gene expression profiling

RNA from DAOY, D425 and ICb-1299 treated or untreated with 2 μM simvastatin was extracted using the Qiagen RNeasy Micro Kit (Qiagen, UK) according to the manufacturer’s descriptions. The concentration of RNA was confirmed using a spectrophotometer (Thermo-Scientific, Wilmington, DE, USA) at the wavelength ratios of A260/230 and A260/280 nm. Libraries were ribosome depleted, pair-end sequenced using the HiSeq 2000 system (Illumina Inc., San Diego, CA, USA) with a read length of 150 bp. Hierarchical clustering was performed using Pearson distance and average linkage after gene-wise standardisation. Differentially expressed probe sets were identified using ANOVA and p-values were adjusted for multiple testing using Benjamini-Hochberg correction in order to control the false discovery rate. An enrichment map was used to visualise results from pathway tests.

### Bioinformatic analysis

Paired-end sequencing ~90 M reads was performed on a primary MB patient cell (ICb1299) versus normal human cerebellar. Libraries were normalised by overall size. The normal human cerebellar (CB) data were taken from GEO: https://www.ncbi.nlm.nih.gov/geo/query/acc.cgi?acc=GSE78564.

The ICb1299 data was taken from NCBI Gene Expression Omnibus database (GSE172363).

### Free cholesterol levels

Cholesterol was measured following the manufacturers instructions using the Amplex™ Red Cholesterol Assay Kit (Catalogue number A12216). Briefly, UW228-2, D425 and D458 cells were washed with PBS two times and cells were sonicated for 5 min. Following this, cells were centrifuged for 5 min at 1000 rpm. 50 µL from the supernatant was seeded in a 96 well plate, followed by a 30-min incubation after addition of reagents. Fluorescence was measured with the FLUOstar Omega microplate reader.

### Total cholesterol level quantification by mass spectrometry

#### Sample preparation

Cholesterol was extracted from DAOY, UW228-2 and D458 cells via a modified Bligh-Dyer Method. In brief, cells were suspended in 1.6 mL of PBS and delipidated with 6 mL of CHCl3:CH3OH (1:2). At this stage d7-Cholestrerol, a deuterated cholesterol used as the internal standard, was added to a concentration of 2 mM in a final lipid extract of 100 μL and left at room temperature for 10 min with vigorous vortex at regular intervals. Each sample was centrifuged at 2400 rpm for 5 min. To the isolated supernatant, 2 mL each of CH3Cl and PBS were added to partition the two generated aqueous-organic phases. The sample was mixed thoroughly and centrifuged at 2400 rpm for 5 min. The lower organic phase was separated and dried under a stream of N2. The lipid liquor was dissolved in 100 μL of methanol, and 1 μL of it was used for analysis by liquid chromatography and high resolution mass spectrometry.

#### Liquid chromatography—mass spectrometry

Extracted lipids were separated using a UPLC BEH C18 2.1 × 50 mm 1.7 μm column (Waters) kept at 40 °C on an ACQUITY UPLC system (Waters Corporation, Milford, MA, USA) coupled to a Synapt qTOF G2Si High Resolution Mass Spectrometer (Waters Corporation, Milford, MA, USA). The mobile phases consisted of (A) mass spectrometry-grade water containing 0.1% formic acid as a modifier, and (B) unmodified mass spectrometry-grade methanol. The following gradient programme was used: 0–2 min, 0% B; 2–6 min, to 85% B, 6 to 6.6 min, to 95% B and kept for an additional at 95% B to 17 min; The gradient returned to the initial isocratic step in 0.6 min after that.

The Synapt G2Si MS was operated in atmospheric pressure chemical ionisation (APCI) in positive ion with the corona needle discharge current set a 1 μAmp. Nitrogen gas flow rates were fixed with the cone gas flow set at 50 L/h and the desolvation gas flow at 600 L/h. A source temperature of 130 °C and a desolvation temperature of 350 °C were applied. Data were acquired using MassLynx v4.1 software (Waters Corporation, Milford, MA, USA) in data independent analysis (DIA) and cholesterol was quantified by MSMS using the following precursor ion > fragment ion transitions: Cholesterol (m/z 369.4 > 161.2) and d7-cholesterol (m/z 376.4 > 161.1).

#### Quantification

An absolute quantification method was developed to quantify cholesterol in cells. Six standards of cholesterol at increasing concentration levels were used to generate a calibration curve. To such purpose, six samples each containing 250, 500, 1000, 2000, 4000, 8000 μM concentrations of cholesterol and a fixed 2000 μM amount of d7-cholesterol, were prepared and analysed by liquid chromatography and mass spectrometry. Cholesterol in each sample was quantified by regression analysis and normalised by cell count in each sample.

#### HRMAS of mevalonate-5-phosphate

Ex vivo tissue was snap frozen after surgery and stored at −80 °C. Prior to use, Trimethylsilylpropanoic acid (TMSP) was added to the samples as a ppm reference. Data was acquired using a 500 MHz Bruker Avance spectrometer (Bruker, Coventry, UK) fitted with a MAS probe. The rotor was spun at 4.8 KHz at a temperate of 278 K. A NOESY pulse sequence was used with 2 s water pre-saturation. Fourier transformed data was imported into MestReNova 9.0.1 software suite (Mestrelab Research, Spain) for metabolite assignment and quantification, with 26 metabolite and 5 lipid macromolecule values being quantified. The concentrations for all the metabolites excluding lipids were determined from the MestReNova analysis in arbitrary units and then were summed to provide a normalisation factor. Mevalonic acid values were reported as the arbitrary value for this metabolite divided by the normalisation factor.

#### 3D-MB spheroid culture

3D spheroids of DAOY, UW228-2 and ICb-1299 MB cells were grown in vitro as previously described by Roper and Coyle [30], suspension cell lines D425 and D458 were cultured following methods described by Hubert et al. [31] in DMEM/F-12 media supplemented with 500 µL N2, 1 mL B-27, 10 µL epidermal growth factor (EGF; 20 ng/ml final concentration), 10 µL heparin (2 µg/µL final concentration) and 5 µL basic fibroblast growth factor (FGF; 10 ng/ml final concentration) all purchased from Sigma (US). Cells were seeded at densities of 250–1000 cells per well in round-bottom ultra-low attachment plates (Nunc, Denmark) and media changed every 2–3 days. Images of spheroids were taken to determine their size before and after treatment using ImageJ software. Also, images were taken to ensure spheroid diameters were the appropriate size (250–350 µm) for treatment and study. The CV% was also calculated to ensure seeding densities were even across plates.

Viability assays were carried out using CellTiter Glo 3D luminescence assay (Promega, US) to assess the effect of simvastatin (2 μM and 5 μM), vincristine (2 nM), etoposide (1 μM), cisplatin (1.98 nM) and combination treatment.

Matrigel extracellular matrix (Corning, US) was used to embed spheroids and assess the effect of simvastatin on invasion of spheroids. Once spheroids were the appropriate size for study, media was removed from ULA plates, Matrigel added and the plate returned to the incubator for 1 h to allow Matrigel to set. 100 μL of neurosphere medium containing simvastatin or chemotherapy was added to the wells and invasion monitored over 72 h. To analyse the extent of spheroid invasion, core spheroid area was subtracted from the area of the spheroid plus invading cells using ImageJ software.

#### Soft Agar Colony Formation Assay

1.5 ml of MB cells were mixed with 0.7% agar (2500 cells/well) and seeded in a 6-well plates previously coated with 1 ml of 0.8% base agar layer [32]. Cells were treated with low doses of simvastatin as indicated. Cells were cultured at 37 °C for 3 weeks. Cells then were stained with 0.05% crystal violet. The images of the plates were analysed using ImageJ software. Each experiment was completed in triplicate and statistical analysis done using Prism software.

#### Xenograft mouse model

All procedures were performed in accordance with licences held under the UK Animals (Scientific Procedures) Act 1986 and later modifications and conforming to all relevant guidelines and regulations. NOD-SCID P4-6 mice were anaesthetised according to standard procedure. Tumour cells (10^5^ cells resuspended in 2 μl sterile PBS) were injected into the right cerebellar hemisphere [28]. Mice were divided randomly into two groups. Animals were housed in sterile IVC cages, monitored thrice weekly and killed humanely when developing neurological signs. At 20 days post-injection mice were treated with simvastatin or placebo, by this time, tumours have established sufficiently to allow for meaningful evaluation of treatment efficacy. Simvastatin belongs to the ‘lipophilic statins’, a sub-class of statins that cross the blood-brain-barrier, and it must be injected every day in the morning because mice are nocturnal animals. Because mice metabolise statins more rapidly than do humans, a dose of 40 mg/kg/day of simvastatin in mice is considered comparable to the maximum dose of 80 mg daily in humans, in terms of serum concentration.

#### Histological examination and immunostaining

Upon development of neurological signs mice were killed, brains and spinal cords were removed and placed in 10% formalin for 24 h, then transferred to PBS. Spinal cords were decalcified using EDTA. Paraffin embedding, coronal sectioning of 3-μm and staining for hematoxylin and eosin (H&E), synaptophysin and human vimentin were performed by UCL IQPath (Institute of Neurology, London, UK). Immunostaining was performed on Ventana Discovery XT instrument an automated staining machines (ROCHE, Burgess Hill, UK) following the manufacturer’s guidelines, using horseradish-peroxidase-conjugated streptavidin complex and diaminobenzidine as a chromogen. The following antibodies were used for histological characterisation: human vimentin (Roche 790-2917, prediluted), Ki67 (Dako, prediluted) and cleaved caspase-3 (Novocastra, prediluted).

For Ki67 and cleaved Caspase 3 images: The number of Ki67 or caspase 3 positive cells was counted in 6 random human vimentin positive brain region. We counted total 4 brain slides from each animal. Data expressed as the average value of positive tumour cells per 100 cells.

Metastatic tumour load is the quantification of tumour area shown as vimentin-positive cells in the section.

#### Generation of DAOY resistant cells to vincristine

Following an initial dose-response test of DAOY cells to varying concentrations of vincristine, a sub-optimal dose was used to treat DAOY cells in culture and select for a resistant population. Resistance was confirmed via generation of concentration-response curves of resistant and non-resistant DAOY cells in the presence of vincristine (0.1–20 nM), using CellTiter Glo 2D luminescence viability assay (Promega, US).

#### Image capturing and analysis

Histological slides were digitised on a LEICA SCN400 scanner (LEICA UK) at 40× magnification and 65% image compression setting, and images were stored on Slidepath Digital Image Hub (Leica Microsystems).

### Statistical analysis

All results are expressed as mean values ± SD or ±SEM of at least three independent experiments. The unpaired Student’s t-test and analysis of variance (one-way ANOVA) were used to assess significant differences between results. P-values of <0.05 were considered statistically significant.

## Results

### A high HMGCR expression with low cholesterol level signature is characteristic of G3/G4-MBs

**Fig. 1:**
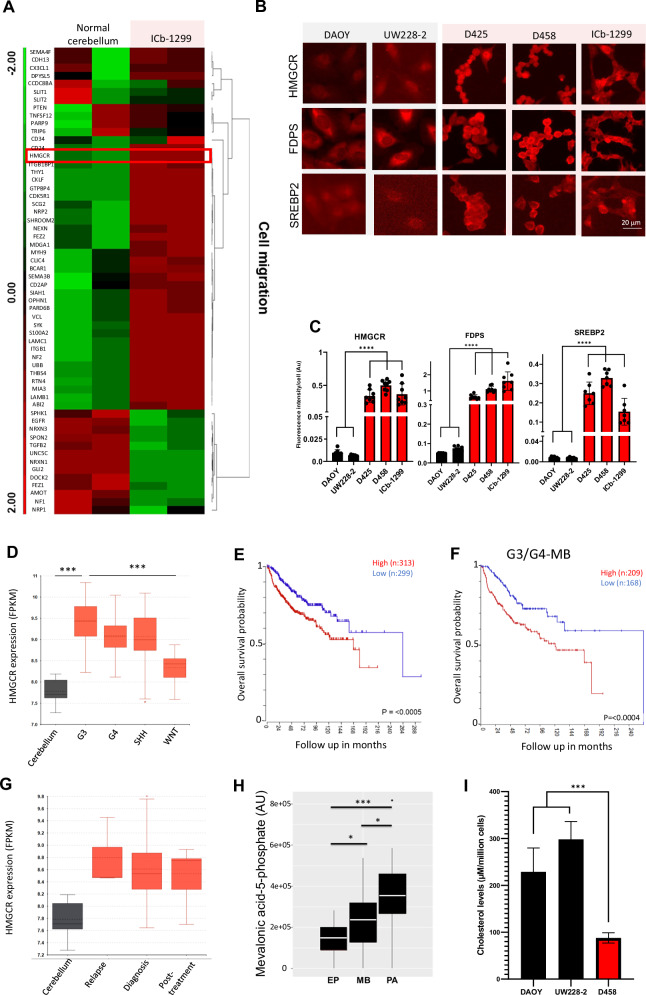
The mevalonate pathway is upregulated in aggressive MBs, highlighting a potential therapeutic target. **A** Heat map representation of the most significantly up-regulated or down-regulated genes in ICb-1299 cells vs normal cerebellum. Genes involved in cell migration were plotted. Genes were identified using Molecular Signatures Database (MSigDB) and plotted to highlight differences in expression of three z-scores or greater between the two groups. (Red) Relative up-regulation; (green) relative downregulation. **B** Representative image of an immunofluorescence (IF) analysis performed in DAOY, UW228-2, D425, D458 and ICb-1299 cells using antibodies against HMGCR, FDPS and SREBP2. Scale bar = 20 µm. **C** Histogram shows mean fluorescence intensities from three independent IF experiments in DAOY, UW228-2, D425, D458 and ICb-1299 cells performed with GraphPad Prism for HMGCR, FDPS and SREBP2. One-way ANOVA performed with Tukey’s post-Hoc comparisons, where * = P < 0.05, ** = P < 0.01, *** = P < 0.001 and **** = P < 0.0001. **D** Box plot representation of HMGCR expression levels (FPKM [fragments per kilobase of transcript per million mapped reads]) in G3, G4, SHH and WNT Medulloblastoma derived from Pfister cohort with 223 patients. Normal cerebellum samples were derived from Roth cohort with 9 patients. **E** Kaplan–Meier overall survival curve based on high and low HMGCR expression levels in MBs tumours derived from Cavalli cohort with 763 patients. Tumours with high HMGCR expression showed decreased survival (P < 0.0005). **F** Kaplan–Meier survival curve based on high and low HMGCR expression levels in G3/G4-MBs tumours derived from Cavalli cohort. Tumours with high HMGCR expression showed decreased survival (P < 0.0004). **G** HMGCR expression levels (FPKM) was assessed using Delattre dataset to compare between tissue taken at diagnosis, post-treatment and relapse (diagnosis: n = 46, post-treatment: n = 8, relapse: n = 3). Normal cerebellum samples were derived from Roth cohort with 9 patients. Welch ANOVA test with Dunnett’s T3 multiple comparisons post-hoc test. **H** Metabolite profiles of ependymoma (EP, n = 16), medulloblastoma (MBs, n = 24) and pilocytic astrocytoma (PA, n = 20) using HR-MAS nuclear magnetic resonance spectroscopy. Kruskal-Wallis analysis shows mevalonic acid-5-phosphate content is significantly different across EP, MBs and PA. **I** Mass spectrometry analysis of soluble cholesterol levels (µM/million cells) in DAOY, UW228-2 and D458 cells.

Metabolic reprogramming is a common oncogenic adaptation to support the rapid growth and metastasis of cancer cells. To dissect which metabolic pathways support cell migration in G3/G4-MBs tumours, we performed a differential gene expression analysis in ICb-1299 (primary human G3/G4-MBs) versus normal cerebellar cells. RNA sequencing (RNA-seq) analysis revealed that a large number of genes involved in cell migration were differentially expressed in G3/G4-MBs (Fig. 1A). We therefore focused our attention on genes that were up regulated in ICB 1299 cells, as this aligned with our goal of identifying genes that may drive the metastatic behaviour of G3/G4 MBs.

*HMGCR* was the most upregulated metabolic gene (>2.0 fold change; P < 0.05) and was selected for validation in a range of human patient derived primary MBs cells and cell lines (Fig. 1A). The highest HMGCR levels were observed in primary G4-MB cells (ICb-1299) and G3-MB cells (D425 and D458), while the lowest levels were found in SHH-MB cells (DAOY and UW228-2) (Fig. 1B, C). These SHH cells harbour p53 mutations but lack MYC amplification, a characteristic associated with a generally better prognosis compared to MYC-amplified SHH tumours [3, 33].

To explore the deregulation of the MVA pathway in greater depth, FDPS (Farnesyl Diphosphate Synthase), another enzyme in the MVA pathway, and Sterol Regulatory Element-Binding Protein (SREBP)-2, which has been shown to transcriptionally regulate *HMGCR*. IF analysis confirmed that FDPS and SREBP2 were highly expressed in G3-MB and G4-MB cells compared to SHH-MB cells (Fig. 1B, C) in keeping with the results of the RNA-seq data. Importantly, positive correlation between *HMGCR* and *SREBP2* was observed (Supplementary Fig. 1A), indicating that cholesterol homoeostasis is maintained by the XBP1/SREBP2/HMGCR axis [34].

To evaluate the clinical significance of HMGCR, we compared expression levels in MB patient data sets. Compared to the normal cerebellum, which shows low HMGCR expression, paediatric brain tumours such as medulloblastoma, ependymoma, paediatric glioma and DIPG, exhibited significantly increased *HMGCR* expression, with the highest levels observed in medulloblastoma (Supplementary Fig. 1B). This high expression of *HMGCR* is conserved in different MB datasets (Supplementary Fig. 1C). Examination of subgroup-specific expression levels also revealed significant upregulation of *HMGCR* in G3-MBs and low expression in normal cerebellum and WNT subgroup (Pfister (n:223) and Roth (n:9) data set, Fig. 1D), which agreed with the results of the RNA-seq and IF data. Also, Kaplan–Meier survival analysis demonstrated that patients with high levels of *HMGCR* expression had a significantly reduced overall survival compared to MB patients with low expression levels (Cavalli data set, n = 763, Fig. 1E). Importantly, high *HMGCR* expression significantly correlated with poor patient survival in the G3-MB and G4-MB subgroups but not in WNT or SHH subgroups (Cavalli data set, n = 763, Fig. 1F, Supplementary Fig. 1D). This suggests that expression of *HMGCR*, could represent a negative prognostic marker for overall survival in MB patients. No significant differences in *HMGCR* gene expression were observed between diagnosis, relapsed group, or post-treatment group, suggesting it does not have a role in the immediate response to treatment or it is not required for relapse (Fig. 1G).

We next confirmed these results by measuring mevalonic acid-5-phosphate, an intermediate precursor of the MVA pathway, by conventional HR-MAS NMR spectroscopic analyses in paediatric cerebellar tumours. MBs (n:24), Ependymoma (EP, n:16) and Pilocytic Astrocytoma (PA, n:20) were selected for analysis on the basis that they account for 95% of paediatric brain tumours [35]. Importantly, these malignancies have very different growth behaviour, where most MBs and EPs are high grade (metastatic and fast growing) while PAs are very slow growing tumours that rarely spread. Interestingly, mevalonic acid-5-phosphate content in fast growing tumours (MBs and EPs) was significantly lower than in PAs indicating high demand of the MVA pathway intermediates in aggressive tumours (Fig. 1H).

The MVA pathway produces many intermediates that are crucial for cell division and migration such as cholesterol, farnesyl phosphate and Coenzyme Q10. Therefore, to dissect if cholesterol was the critical end product, we analysed cholesterol content in DAOY, UW228-2 and D458 cells via mass spectrometry (Fig. 1I, Supplementary Fig. 1E). Our data shows that D458 cells have significantly reduced soluble cholesterol levels compared to DAOY and UW228-2 (Fig. 1I). This was further validated by measuring free cholesterol in DAOY, UW228-2 and D458 (Supplementary Fig. 1F). Reduced free cholesterol levels were detected in D458 cells vs DAOY and UW228-2. Taken together, these data indicate that G3-MB tumours upregulate the MVA pathway for synthesis of isoprenoid intermediates rather than cholesterol per se.

### The mevalonate pathway contributes to the biological functions of Rho-GTPases signalling pathway in G3/G4-MBs

**Fig. 2:**
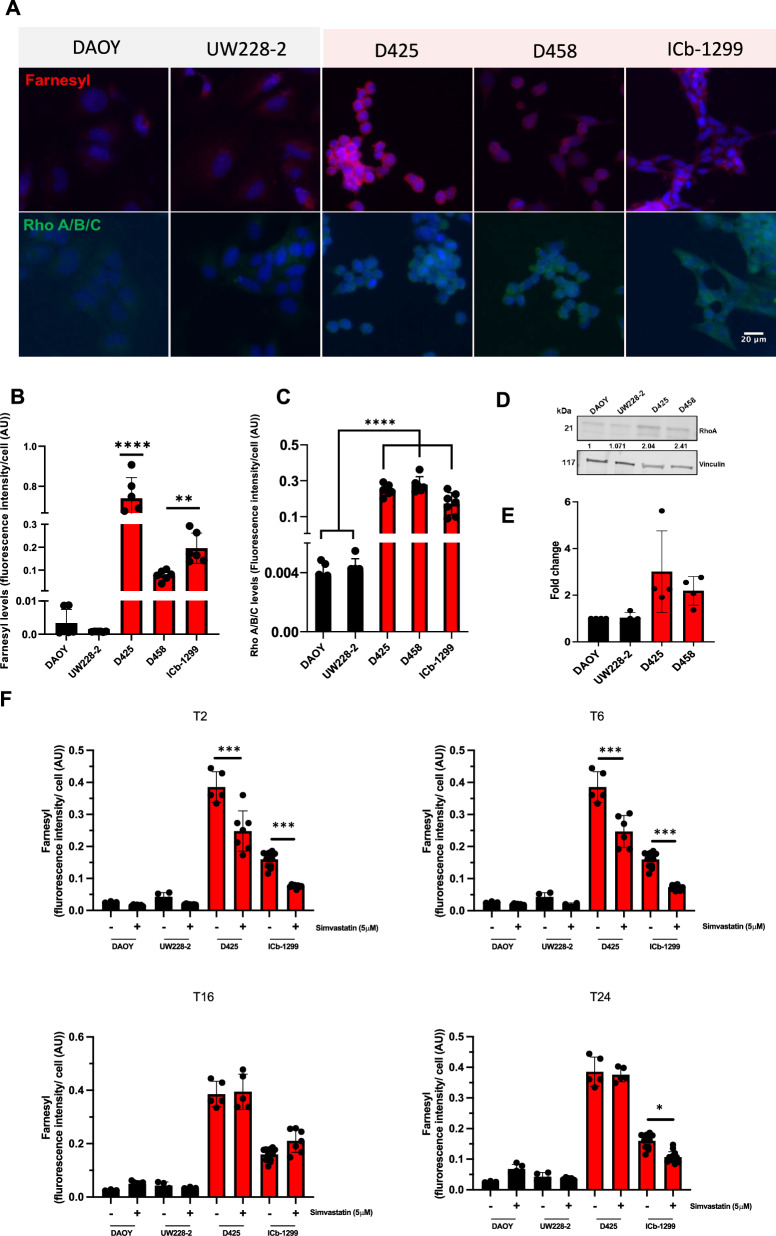
Rho-GTPase signalling supports MB cell migration. **A** Representative photographs of immunofluorescence analysis performed in DAOY, UW228-2, D425, D458 and ICb-1299 cells using antibodies against anti-Farnesyl and RhoA/B/C proteins. Nucleus was stained with DAPI (blue). Scale bar = 20 µm. Quantification of mean fluorescence intensities from 3 independent experiments of anti-Farnesyl (**B**) and Rho A/B/C (**C**) in DAOY, UW228-2, D425, D458 and ICb-1299 cells performed with GraphPad Prism. One-way ANOVA performed with Tukey’s post-Hoc comparisons, where ** = P < 0.01 and **** = P < 0.0001. **D**, **E** Western blotting showing Rho A levels in SHH-MB (DAOY, UW228-2) vs G3-MBs (D425, D458) cells. Vinculin was used as a loading control. Fold change in protein levels between SHH cells and G3/G4 MBs cells is displayed. **F** Quantification of mean fluorescence intensities from three independent experiments of anti-Farnesyl in DAOY, UW228-2, D425, D458 and ICb-1299 cells after a single treatment with 5 μM simvastatin. Simvastatin treatment was performed for 2, 6, 16 and 24 h.

The MVA pathway plays a crucial role in producing vital cellular components such as geranylgeranyl pyrophosphate and farnesyl pyrophosphate, for mitochondria function, cell cycle and cell migration (Supplementary Fig. 1A). Our data shows that G3/G4-MB cells significantly express more protein farnesylation than SHH-MB cells (Fig. 2A, B). Notably, the farnesylation modification of Rho-GTPases plays a pivotal role in orchestrating cellular migration, and is crucial for ensuring their appropriate subcellular localisation to the plasma membrane, a prerequisite for their biological activity [18]. IF analysis revealed increased expression of RhoA/B/C in G3/G4 MBs versus SHH-MBs cells (Fig. 2A, C). To corroborate this finding, western blot analyses were conducted to detect the expression activity of the Rho-GTPase family, encompassing RhoA/B/C, Rac, and Cdc42 proteins. The results indicated increased expression of the Rho-A/B/C family in G3/G4-MB cells compared to their SHH-MB counterparts (Fig. 2C, D). We also observed that Rac1 and Cdc42 protein levels were low compared to Rho levels in MB cells, prompting us to investigate if changes in culture condition, from 10% to 1% FBS, may influence their relative expression (Supplementary Figure 2B–E). We observed that Rac1 and Cdc42 were more expressed in G3/G4-MBs versus SHH-MBs. Also, changes in culture conditions did not affect Rho-GTPases expression. Taken together these data indicate that the MVA pathway supports G3/G4-MBs migration by activating the Rho-GTPase pathway.

The effect of single low doses of simvastatin, a competitive inhibitor of HMGCR, was tested to evaluate the effect on farnesyl and RhoA/B/C pathway in G3/G4-MBs and SHH-MBs cells. As shown in Fig. 2F, 5 µM simvastatin significantly reduced farnesyl levels in G3/G4-MBs cells at 2- and 6-h post-treatment, no significant effect was observed in SHH-MBs cells. However, this effect was no longer observed at 16- and 24-h post-treatment in G3/G4-MB cells. The initial reduction in farnesyl levels is due to the rapid onset of simvastatin’s inhibitory effects on HMGCR, which has a short half-life of 1 h [36]. Over time, new HMGCR is synthesised and simvastatin is metabolised and eliminated, and its intracellular concentration may decline. Consequently, its inhibitory effect diminishes, allowing farnesyl levels to recover by 16- and 24-h (Fig. 2F).

Importantly, 5 µM simvastatin did not affect Rho levels in MB cells at 6-h post treatment (Supplementary Fig. 2F). Rho protein levels are not significantly affected in SHH and G3/G4-MB subgroups at early time points because the protein has already been synthesised and prenylated prior to simvastatin treatment, the half-life of Rho is typically around 24 h under normal physiological conditions [37]. By 16-h of simvastatin treatment, we observe reduced Rho proteins in G3/G4-MB cells, impairing their localisation and stability (Supplementary Fig. 2F). Importantly, we observed a reduction of Rho at 6-h only in ICb-1299 cells which are a patient derived cell. These data validate that the Rho-GTPases are indirectly linked to the cholesterol pathway through their dependence on the mevalonate pathway to be farnesylated.

### Clinically significant doses of simvastatin trigger transcriptomic changes in SHH and G3/G4-MBs subgroups

**Fig. 3:**
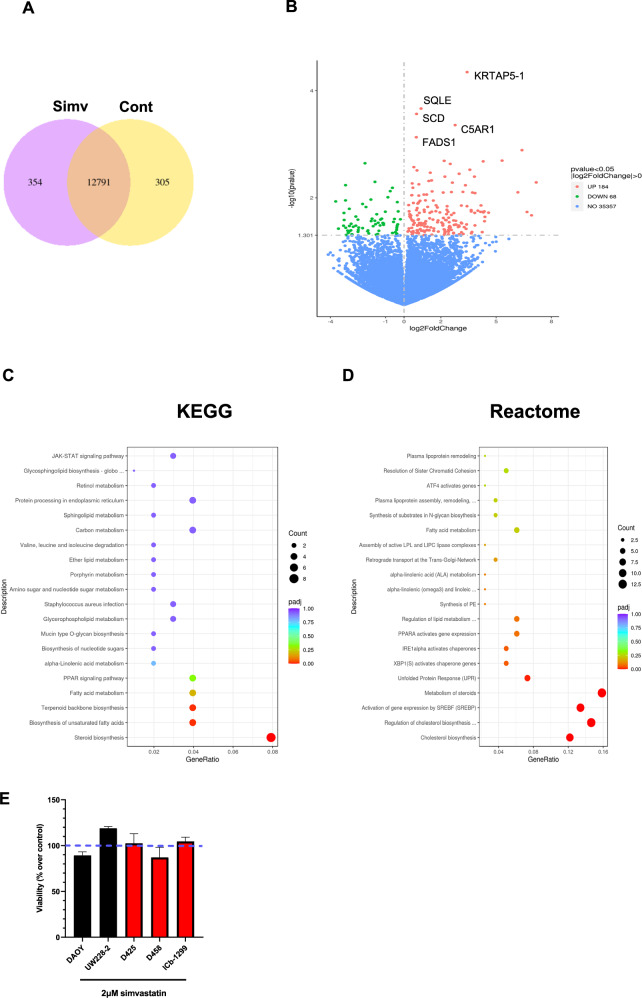
Simvastatin induces significant transcriptomic changes in SHH- and G3/G4-MB cells. **A**–**D** DAOY, D425 and ICb-1299 cells were treated with simvastatin for 72 h before the cells were harvested for RNA-seq library preparation. **A** Venn diagrams of DEGs of shared or not by the ImmuneScore analyses. **B** Volcano plots of significant DEGs (FDR-adjusted p < 0.05, |log2 FC| > 0) between high and low expressed genes. **C** KEGG and **D** GO enrichment analyses (p < 0.05 and q < 0.05). **E** Viability assay of DAOY, UW228-2, D425, D458 and ICb-1299 cells after treatment with 2 µM simvastatin for 72 h. Viability evaluated using CellTiter Glo luminescent assay (Promega).

Previous studies have shown that statins have many effects including antiproliferative, pro-apoptotic and inhibition of migration and invasion [21, 22, 26] that are cancer type and concentration-dependent. In order to investigate the transcriptional alterations induced in MBs by single low doses of simvastatin (2 µM), RNA-seq was performed. DAOY, D425 and ICb-1299 cells were treated with simvastatin for 72 h before the cells were harvested for RNA-seq library preparation. A significant 354 differentially expressed genes were observed between the control and simvastatin treated cells (Fig. 3A). A volcano plot generated from this data shows that 184 genes were upregulated, and 68 genes were downregulated (Fig. 3B). KEGG and Reactome analysis of genes upregulated upon simvastatin treatment shows an enrichment for pathways involved in steroid biosynthesis, unsaturated cholesterol, fatty acid metabolism, cholesterol biosynthesis and cholesterol metabolism (Fig. 3C, D and Supplementary Fig. 3A, B). The enrichment of pathways involved in lipid metabolism suggests a potential compensatory effect in response to simvastatin treatment. Interestingly, the enrichment of SREBP-regulated genes agrees with the hypothesis that the XBP1/SREBP2/HMGCR axis regulates the mevalonate pathway in MBs. No apoptotic pathways were observed to be enriched after 72 h treatment with low doses of simvastatin (2 µM) (Fig. 3C, D), which was further confirmed by viability assays and Annexin V/PI (Fig. 3E, Supplementary Fig. 3C, D). In addition to this, the effect of high-dose simvastatin (10 µM) in MB cells did not induce a noticeable cell cycle arrest, reflecting the lack of enrichment of senescence-associated pathways observed following treatment with low-dose simvastatin (Fig. 3C, D, Supplementary Fig. 3E). In conclusion, these results indicate that clinically relevant doses of simvastatin did not impact cell cycle progression or decrease cell viability of MB cells.

### Low doses of simvastatin reduce cell migration of SHH and G3/G4-MB subgroups

**Fig. 4:**
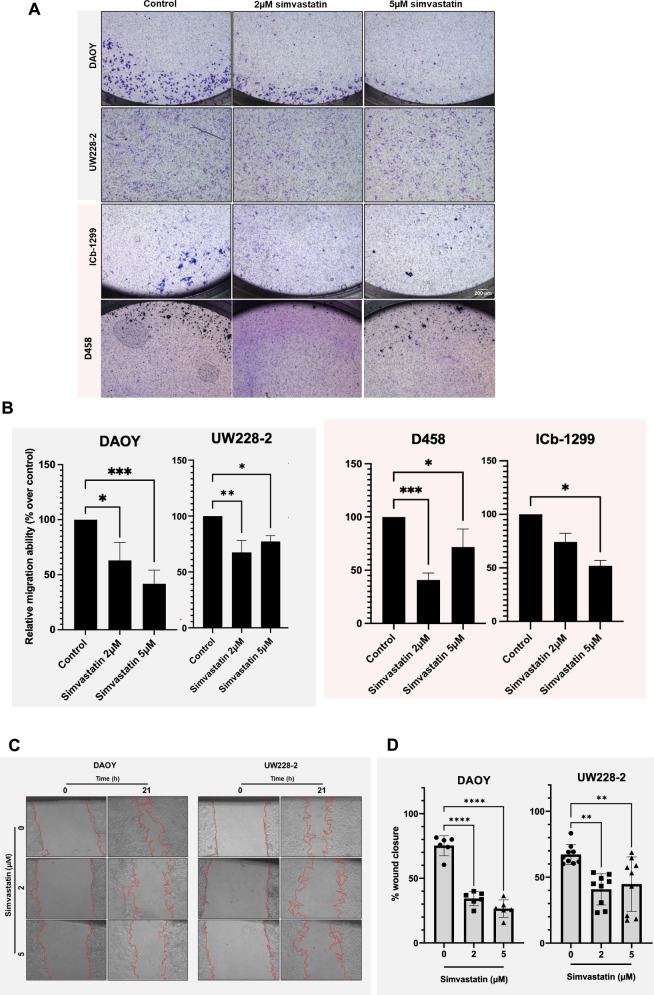
Simvastatin reduced SHH-MB and G3/G4-MB cell migration. **A** Representative microscopic images of DAOY, UW228-2, ICb-1299 and D458 cells that migrated through the transwell. MB cells were treated with 2 and 5 µM simvastatin. **B** Quantification of transwell-migrated cells. Data are shown as relative migration ability from three independent experiments. **C** Scratch assays in adherent SHH cells (DAOY and UW228-2) treated with simvastatin (2 µM and 5 µM). Wound size calculations taken at 0 h and 21 h. **D** Quantification of migrated DAOY and UW228-2 cells carried out using ImageJ software. One-way ANOVA statistical analysis done using GraphPad Prism software.

Given our interest in identifying metabolic pathways driving the metastatic behaviour of MBs (Fig. 1A), we focused on the critical role of the MVA pathway in cell migration, specifically through the farnesylation of Rho-GTPases. Therefore, we validated the role of the mevalonate pathway in MB cell migration with transwell and wound-healing assays. Interestingly, 2 µM and 5 µM simvastatin significantly inhibited G3-/G4-MB and SHH-MB cell migration in a dose-dependent manner (Fig. 4A, B). To strengthen the observation that Rho-GTPases are essential for G3-/G4-MB migration, we used the potent p160 ROCK inhibitor Y-27632. 50 μM Y-27632 significantly reduced D458 cell migration (Supplementary Fig. 4A). In addition to this, the effect of vincristine, an inhibitor of microtubule formation, was tested, which also reduced the migration of SHH-MB cells (Supplementary Fig. 4B, C).

Subsequently, we performed a scratch assay in SHH-MB cells and observed that 2 µM and 5 µM simvastatin significantly reduced the migration of DAOY and UW228-2 cells (Fig. 4C, D). These findings suggest that, regardless of the relative expression levels of RhoA/B/C proteins in MB cells, key downstream effectors play a critical role in migration in SHH-MBs. The GTPase proteins exhibit functional redundancy and their collective activity depends on the activity of prenylated proteins [38, 39].

Taken together, this data shows that clinically significant doses of simvastatin inhibit MB cells migration by reducing prenylation of Rho-family of proteins.

### Low doses of simvastatin suppress anchorage-independent growth in G3/G4-MB and SHH-MBs cells

**Fig. 5:**
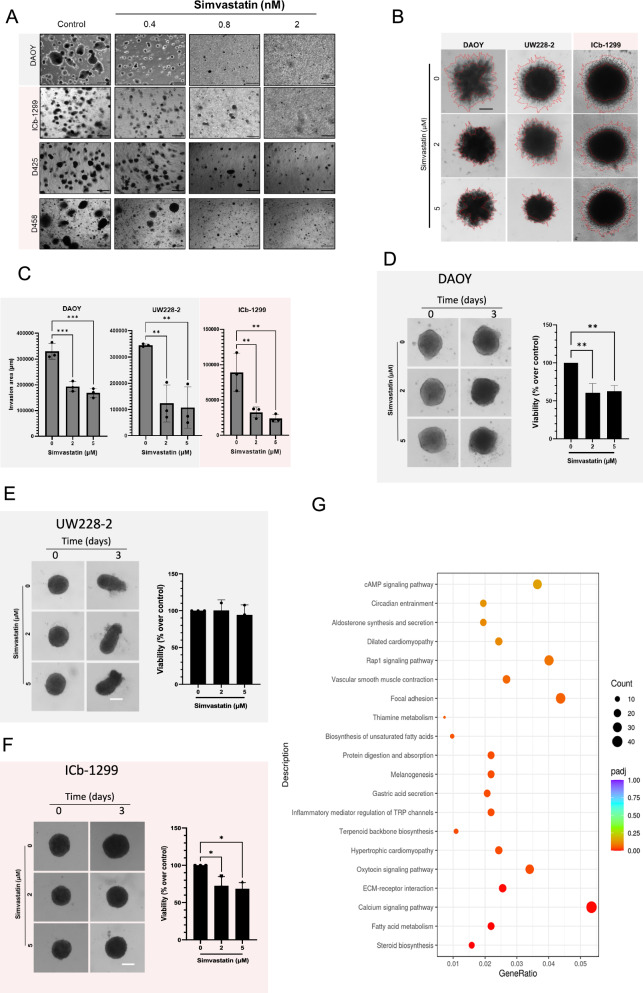
Simvastatin reduced SHH-MB and G3/G4-MB colony formation in soft agar. **A** Representative images of DAOY, ICb-1299, D425 and D458 cells. Colony formation assay shows a concentration dependent decrease in the number of MBs colonies after 0.4, 0.8 and 2 nM simvastatin treatment for 1 month. **B**, **C** Invasion studies of 3D-MB spheroids encapsulated in Matrigel basement membrane with or without presence of simvastatin (2 and 5 µM) for 72 h. **B** Representative image of DAOY, UW228-2 and ICb-1299. **C** Quantification of invasion of DAOY, UW228-2 and ICb-1299 cells carried out using ImageJ software from three independent experiments. Representative images and viability assay of DAOY (**D**), UW228-2 (**E**) and ICb1299 (**F**) Spheroids after treatment with 2 and 5 µM simvastatin from 3 independent experiments. ** = P < 0.01. **G** DAOY, D425 and ICb-1299 cells were grown in 2D or 3D culture conditions before the cells were harvested for RNA-seq library preparation. KEGG enrichment analyses is shown (*p* < 0.05 and *q* < 0.05).

To validate the effect of low doses of simvastatin on G3/G4-MB and SHH-MB cells mediated by the Rho family of proteins, a soft-agar colony formation assay was performed. This method assesses anchorage-independent growth, which depends on cytoskeletal organisation, cell adhesion, proliferation and survival—all processes regulated by the Rho-GTPase pathway, which play a critical role in cytoskeletal regulation [38, 40]. MB cells were cultured for 1 month, with different concentrations of simvastatin added every other day (Fig. 5A and Supplementary Fig. 5A, B). Clinically relevant doses of simvastatin ranging from 0.4 to 2 nM were used for the duration of 4 weeks in this study. Interestingly, a significant difference in the number and size of the colonies formed was observed (Fig. 5A and Supplementary Fig. 5A, B). Similar results were obtained in G3/G4-MB and SHH-MB cells. These data indicate that simvastatin reduced colony formation ability and anchorage-independent growth of G3/G4-MB and SHH-MB cells.

Invasion of surrounding normal tissues is a key marker of malignancy. Therefore, a 3D invasion assay was performed in DAOY, UW228-2 and ICb-1299 spheroids. Spheroids were embedded in Matrigel and simvastatin (2 and 5 µM) was added or not for 3 days. A 70% reduction in the degree of invasion was observed in all 3D spheroids after treatment with simvastatin (Fig. 5B, C, Supplementary Fig. 5C).

To determine if the lack of 3D invasion after simvastatin treatment was due to cell death, we performed cell viability assays at 3- and 5-days post treatment. After 3 days of simvastatin treatment, we observed that DAOY spheroids have a reduction of 50% in cell viability (Fig. 5D), UW228-2 do not show any cell death (Fig. 5E) and ICb-1299 spheroids showed a 30% reduction in viability (Fig. 5F). At 5 days of simvastatin treatment the behaviour of the spheroids was similar between DAOY and ICb-1299 showing a significant reduction in cell viability and UW228-2 spheroids were found to be partially resistant to simvastatin treatment (Supplementary Fig. 5C, D, E).

To understand the high sensitivity of 3D spheroids to simvastatin treatment, RNA-seq was performed on 2D versus 3D cultures. DAOY, D425 and ICb-1299 cells were cultured either as 2D monolayers or as spheroids. The top enriched KEGG pathways were steroid biosynthesis, fatty acid metabolism and calcium signalling suggesting that the change in MB cells culture condition can affect the dependency of metabolic pathways, with MB spheroids exhibiting greater addiction to lipid metabolism (Fig. 5G and Supplementary Fig. 5F) In conclusion, these results indicate that clinically relevant doses of simvastatin reduce MBs cell invasion, by reducing cell movement or by cell death induction. Also, it shows that MB growing as spheroids cells are cholesterol addicted.

### Simvastatin reduces G4-MB tumour growth and spinal cord metastasis in vivo

**Fig. 6:**
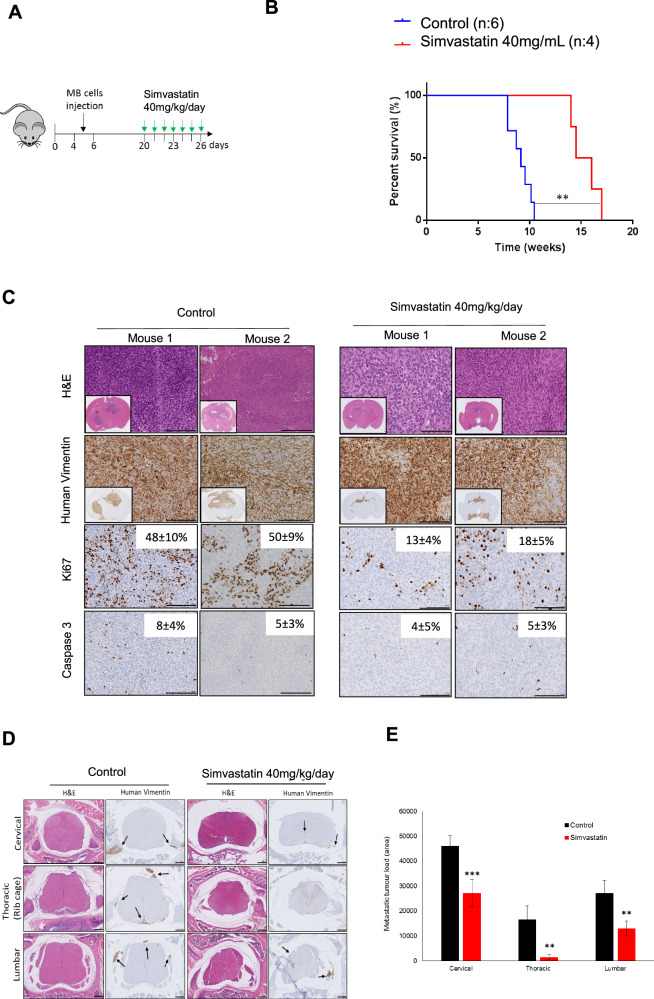
Low doses of simvastatin result in significantly improved survival in an orthotopic MBs xenograft model. **A** ICb-1299 human primary cells were injected into the cerebellum of newborn NOD-SCID mice, and mice were divided into control (n = 6) and simvastatin (n = 4). At 20 days old, 40 mg/Kg/day simvastatin was injected i.p in the morning every day in the simvastatin group. **B** The survival of the mice is plotted over time (log-rank test) (**) P < 0.001. **C** Histology of the MBs tumours treated or not with 40 mg/kg/day simvastatin. Representative photographs of H&E showing typical MBs tumour morphology at 10 weeks. Staining with human vimentin confirms their origin. Representative bright-field images are shown for Ki-67 and cleaved caspase-3. Quantification is shown as the mean of positive cells per high-power field. Bar, 50 µm. **D** Histology of the spinal cord of the mouse treated or not with 40 mg/kg/day simvastatin. Representative photographs of H&E showing typical morphology at endpoint experiment. Staining with human vimentin confirms their origin. Bar, 50 µm. **E** Quantification of the tumour load. (**) P < 0.001; (***) P < 0.0001. H&E: haematoxylin and eosin.

Next, validation of in vitro results in an in vivo mouse model of MBs was carried out. G3/G4 MB cells (ICb-1299) were injected into the cerebellum of newborn mice, with everyday treatment with simvastatin commencing from 20 days of age until neurological symptoms occurred (Fig. 6A). Because mice metabolize statins more rapidly than humans, a dose of 40 mg/kg/day of simvastatin in mice is considered comparable to the maximum dose of 80 mg daily in humans, in terms of serum concentration [41]. Daily 40 mg/kg/day intraperitoneal injections showed that simvastatin treatment is well tolerated in vivo (Supplementary Fig. 6A).

Animals treated with simvastatin displayed a significant increase in survival time compared with saline treated control (Fig. 6B), in keeping with our previous observation that simvastatin reduced colony formation of MB cells. Injection of lCb-1299 into the cerebellum of newborn NOD-SCID mice rapidly induced tumours with histological and immunohistochemical features of aggressive MBs (Fig. 6C). A significant reduction in cell proliferation was detected (Ki67 staining) with no significant apoptosis induction (cleaved caspase-3) in animals treated with simvastatin (Fig. 6C). These results agree with previous in vitro data, in which simvastatin leads to reduced colony formation of MB cells.

Intracranial and spinal cord metastatic disease spread is the major cause of treatment failure and mortality among MB patients. We next asked whether simvastatin treatment leads to reduced metastatic disease in the spinal cord since it inhibits MB cells migration in our in vitro models. A series of coronal sections of the entire spinal cord, cervical, thoracic and lumbar (Supplementary Fig. 6B) of xenograft mouse was examined for cytology (H&E staining), and presence of MB tumour cells was confirmed by staining for expression of human vimentin (Fig. 6D). Strikingly, it was found that simvastatin treatment significantly reduced metastatic load in the spinal cord of mice (Fig. 6D, E). This effect by simvastatin was observed throughout the entirety of the spinal cord (Fig. 6D, E). In summary, we found that simvastatin treatment reduced MB cells proliferation and migration of aggressive MB cells to the spinal cord.

### Additive effect of simvastatin plus etoposide or simvastatin plus vincristine in MBs

**Fig. 7:**
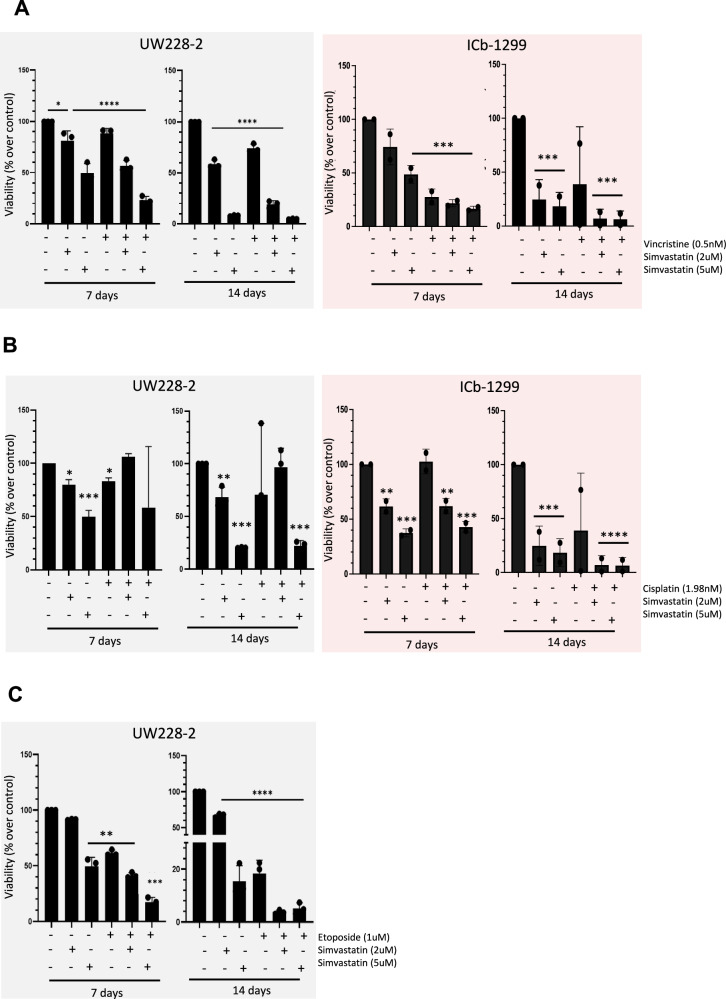
Additive effect of simvastatin plus chemotherapeutic drug treatment in SHH- and G3/G4-MB cells. Viability of UW228-2 and ICb-1299 spheroids after 7- day and 14-day treatment with simvastatin (2 and 5 µM), with or without 0.5 nM vincristine (**A**), 1.98 nM cisplatin (**B**) or 1 μM etoposide (**C**). One-way ANOVA performed with Tukey’s post-hoc comparisons: (*) P < 0.05; (**) P < 0.001; (****) P < 0.0001.

Combination therapies are widely used for the treatment of the most dreadful diseases, such as cancer or acquired immune deficiency syndrome. Chemotherapy drugs such as vincristine, cisplatin and etoposide are commonly used to treat MBs patients [9]. Therefore, we set to test if simvastatin has any synergic, additive or antagonistic effect with different chemotherapy drugs. UW228-2 and ICb-1299 spheroids were chosen because of their high resistance to simvastatin treatment (Fig. 5D, F, Supplementary Fig. 5C, E) or chemotherapy drugs treatment [27]. Spheroids were treated with 2 or 5 μM simvastatin alone or in combination with vincristine (0.5 nM), cisplatin (1.98 nM) or etoposide (1 μM), for 7 and 14 days. We observed that simvastatin has an additive effect with all the chemotherapy drugs tested (Fig. 7A–C and Supplementary Fig. 7A). A significant reduction of ATP levels was observed after 7 and 14 days of the combination treatments (Fig. 7A–C and Supplementary Fig. 7A). These results demonstrate that simvastatin exhibits an additive, rather than antagonistic effect, with chemotherapy drugs used to treat MB- highlighting its promise for future potential use in combination therapy in patients.

Following this, we decided to investigate whether simvastatin could be a viable treatment option for resistant-MB tumours, which currently have very limited therapeutic options. MBs-resistance to treatment is driven by a combination of cellular and molecular mechanisms that enhance the tumour’s ability to evade treatment. To model resistance, we generated vincristine-resistant DAOY-MB cells by gradually exposing DAOY cells to increasing concentrations of vincristine. Our resistant cells exhibited a significantly higher IC50 for vincristine (17.2 nM) compared to the control cells (3.8 nM) (Supplementary Fig. 7B). Interestingly, when we tested the sensitivity of both resistant and control cells to simvastatin, they displayed similar IC50 values (1.54 µM and 1.48 µM, respectively). These results suggest that simvastatin could potentially serve as an effective treatment for certain resistant-MB tumours (Supplementary Fig. 7B).

In summary, this data reveals that clinically relevant doses of simvastatin reduced MB tumour anchorage and metastasis by impairing protein prenylation and activation of the Rho-GTPase pathway, all of which have been previously implicated as key drivers of tumour growth and metastasis in cancer cells.

## Discussion

In the last decade, the focus upon cancer metabolism has shifted from a mode of distinguishing malignant cells to its key hallmark role in transformation [42]. A key part of this involves genetic mutations that de-regulate certain metabolic pathways which in turn encourage progression of cancer cells [43]. There are several metabolic adaptations that have been described as advantageous in cancer cells, such as the use of fatty and amino acids as alternative energy sources to support cancer cell growth and progression [44]. Therefore, the potential use of lipid inhibitors as anti-cancer treatment is ready to be explored.

MBs are highly malignant paediatric brain tumours, and some of these tumours are very difficult to treat due to the presence of metastasis at diagnosis [45]. Although the majority of children with MBs can be cured, the burden of complications for survivors is great due to the intensity of therapy given to ensure complete destruction of the tumour [46]. Recent studies have highlighted the various anti-tumour effects of statins on bladder, lung, gastric, breast cancers and osteosarcoma [22, 27, 47–49], ranging from cell cycle arrest and apoptosis to a reduction in cell migration. The upregulation of the MVA pathway observed in G3/G4-MBs is similar to previous studies in breast, lung and glioblastoma tumours, supporting the idea that upregulation of this pathway is a common metabolic adaptation of aggressive tumours.

This study demonstrates that low doses of simvastatin, a drug used to inhibit cholesterol biosynthesis with the ability to cross the BBB, can reduce migration of MB cells and impair protein prenylation through farnesylation and geranylgeranylation. Statins induced antitumor effects in cancer cell and preclinical animal models, by inducing apoptosis, cell cycle arrest and inhibition of cell proliferation and invasion.

It has been shown that aggressive tumours are characterised by a high capacity to metastasise, resulting in constant fluctuations in nutrients [50]. Therefore, upregulation of the MVA or de novo lipogenesis presents a reliable source of energy, while uptake of other extracellular lipid sources (exosomes) and amino acids (glutamine) fuels proliferation of cancer cells [51–53]. In addition to this, some cancer cells are able to engulf necrotic cell debris and even entire living cells when energy resources become low, in order to maintain their metabolism for cell proliferation [51]. This study demonstrates that the MVA pathway supports migration of MB cells.

Cell migration is important for development and function of the central nervous system; however, it is also associated with many diseases such as inflammation and cancer cell metastasis [45]. Migration of cells is reliant on modelling and remodelling of actin filaments which form the lamellipodia, filopodia and invadopodia needed for cell movement [38]. These processes also incorporate the involvement of Rho-GTPases. For Rho-GTPases to become active, post-translational modifications need to occur, ensuring adequate cell movement [54]. These Rho proteins can be modified covalently through addition of farnesyl/geranylgeranyl groups and/or a palmitoyl lipid [39, 55]. These modifications allow trafficking of Rho proteins to cell membranes, allowing normal biological activity, therefore, inhibition of the MVA will reduce prenylation levels and in-turn reduce the levels of attachment of Rho-GTPases to the cell membrane. Here, we show that low doses of simvastatin are associated with reduction in MB cell migration, similar to a previous study where simvastatin reduced migration of breast cancer cells [48]. Because Rho-GTPases are responsible for regulation of invasion as well as migration, as shown previously in glioblastoma [20], interference with the activation of these migratory proteins consequently affects the invasive capacity of cells. 3D-MB spheroids treated with simvastatin showcased a marked decrease in invasion, suggesting once again that MB tumours rely on the activation of these migratory proteins to facilitate invasion into the surrounding microenvironment, allowing metastasis of the disease. The ability of simvastatin to decrease invasion as well as migration provides further evidence for the use of statins as therapy for MBs.

Interestingly, as well as the ability of simvastatin to decrease the invasive capacity of 3D spheroids in vitro, it appears in the SHH-MB subgroups (DAOY and UW228-2) that even low doses of simvastatin can reduce viability (Fig. 5), compared to in 2D models where there is very little effect on cell viability (Fig. 3E). This could indeed be a result of increased reliance on lipid metabolism in a 3D structure, which then makes blockage of MVA progression highly detrimental to solid tumours. This phenomenon needs to be investigated further, and has already been highlighted by some researchers in SHH MB and GBM [56–59].

Combination therapies that present with synergistic or additive effects are a valuable tool for the treatment of many diseases. We have demonstrated that simvastatin has an additive effect with conventional chemotherapy drugs used to treat MB patients. Also, we have shown that simvastatin could be used for the treatment of some resistant MB tumours, which have very limited treatment options.

The clinically relevant doses of simvastatin used here showcase that these doses are sufficient to reduce metastatic spread of G3/G4-MBs cells to the spinal cord in vivo, as well as decreasing invasion in a 3D in vitro model, in part through reducing protein prenylation. Metastasis is a hallmark of late-stage disease and responsible for the highest number of cancer-related fatalities. We recognise that our model utilising immunocompromised mice had limitations, and future studies incorporating full non-immunocompromised animals as well as integrating other formats of tumour imaging would further strengthen the interpretation of treatment effects and tumour microenvironment role. We demonstrated here that MB cells depend on the MVA to support migration and therefore suggest that the already FDA and MRHA-approved drug simvastatin could potentially be used as an adjuvant treatment option for MB patients.

## Supplementary information

Supplementary Figure 1, Supplementary Figure 2, Supplementary Figure 3, Supplementary Figure 4, Supplementary Figure 5, Supplementary Figure 6, Supplementary Figure 7

Supplementary Figure legends

## Data Availability

All raw data generated in this study are available upon request from the corresponding author.
