# Supplementary material for: Simvastatin suppresses spinal cord metastasis of medulloblastoma at clinically significant doses
Source: Cell Death Dis. 2025 Jul 15;16(1):527. doi: 10.1038/s41419-025-07829-0 (PMC12263873; doi:10.1038/s41419-025-07829-0)
Supplement: Supplementary file 1 — Supplementary Figure 1, Supplementary Figure 2, Supplementary Figure 3, Supplementary Figure 4, Supplementary Figure 5, Supplementary Figure 6, Supplementary Figure 7 [file 41419_2025_7829_MOESM1_ESM.pptx]

## Slide 1
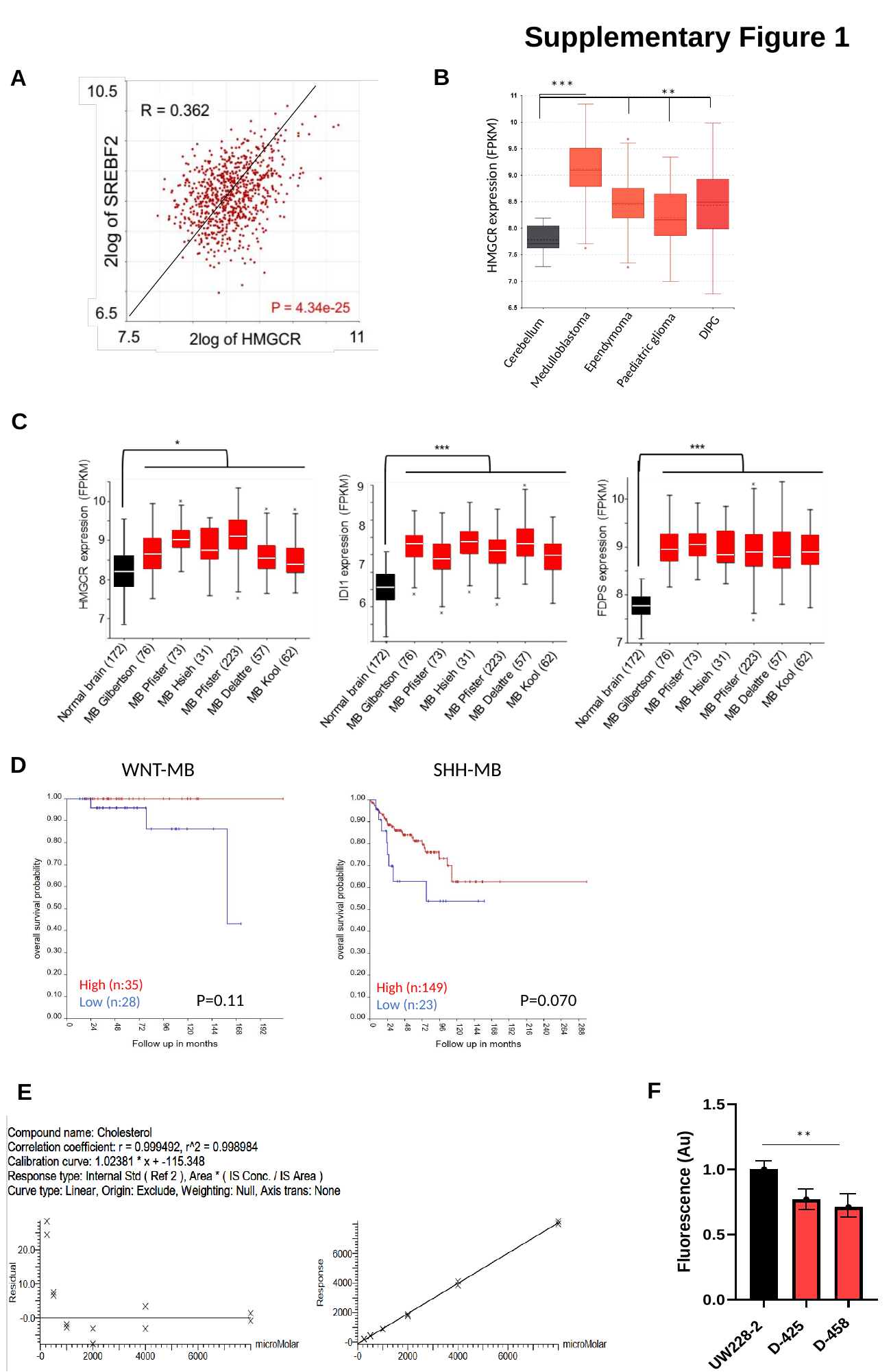

Supplementary Figure 1
B
A
***
**
HMGCR expression (FPKM)
DIPG
Ependymoma
Medulloblastoma
Paediatric glioma
Cerebellum
C
D
WNT-MB
SHH-MB
High (n:35)
Low (n:28)
High (n:149)
Low (n:23)
P=0.070
P=0.11
F
**
E

## Slide 2
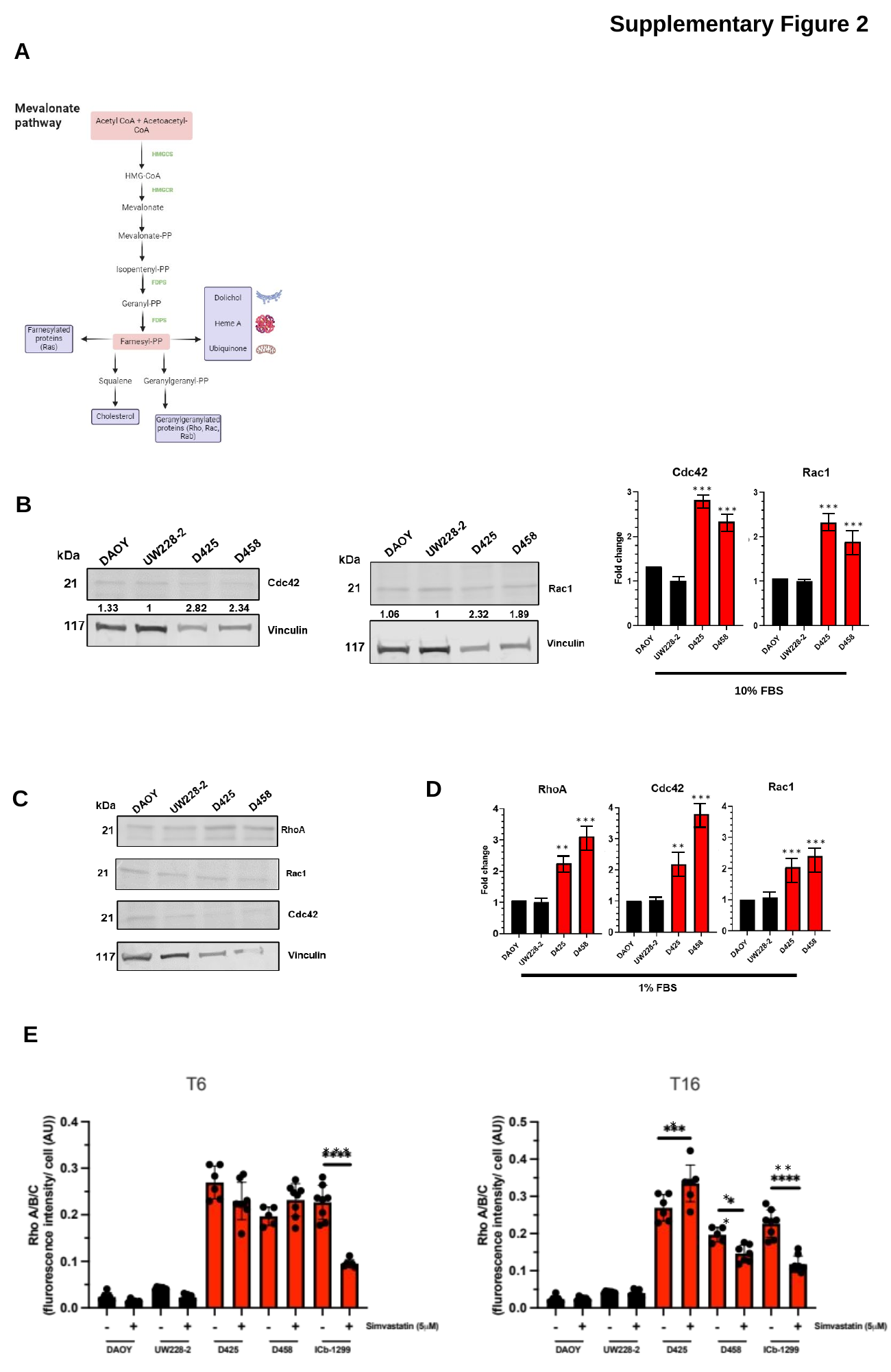

Supplementary Figure 2
A
***
B
***
***
***
10% FBS
D
C
***
***
***
**
**
***
E
*
***
**
*
*

## Slide 3
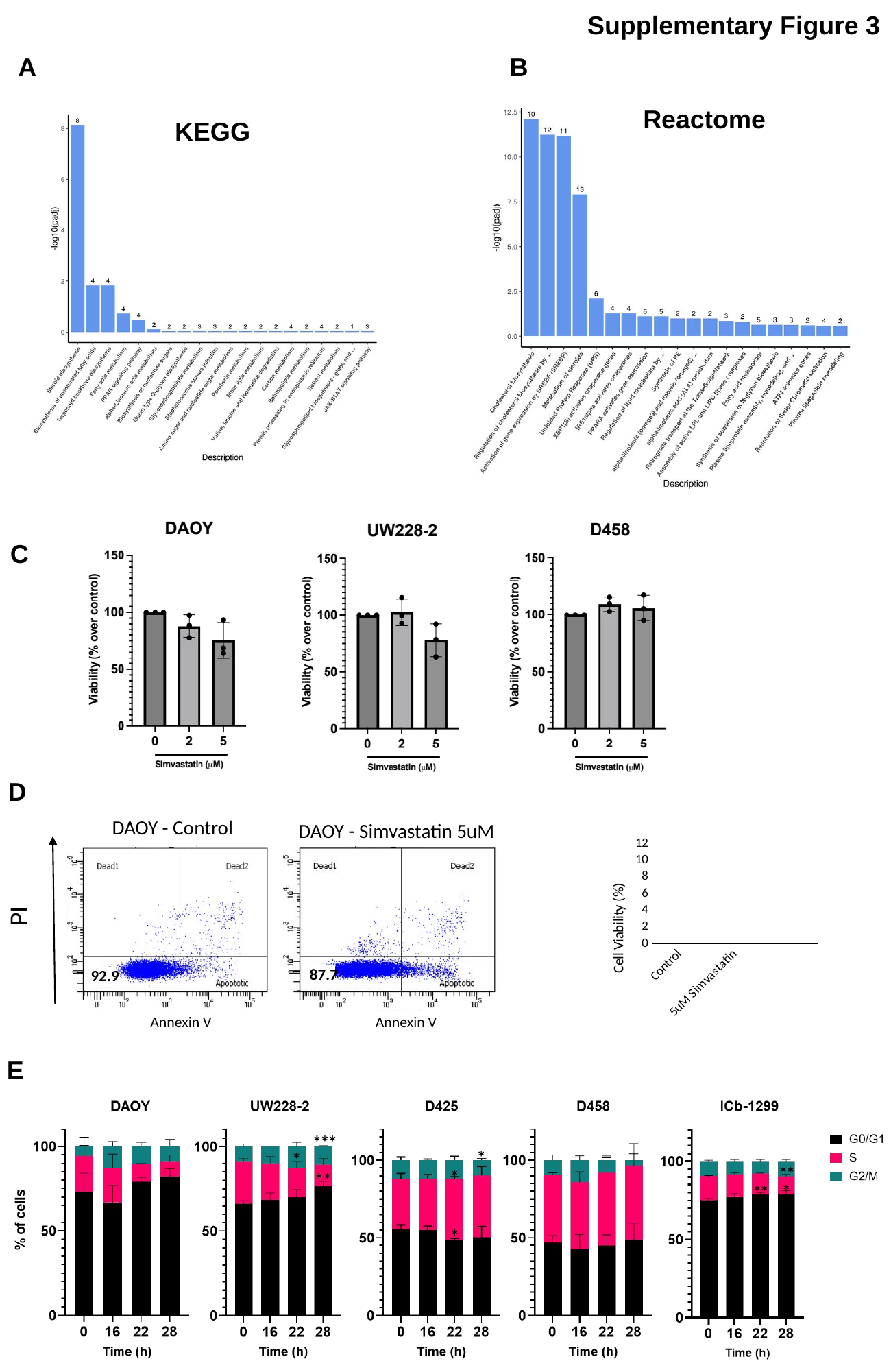

Supplementary Figure 3
A
B
Reactome
KEGG
C
D
DAOY - Control
DAOY - Simvastatin 5uM
PI
87.7
92.9
Annexin V
Annexin V
### Chart
| Category | |
|---|---|
| Control | 92.0 |
| 5uM Simvastatin | 87.0 |E

## Slide 4
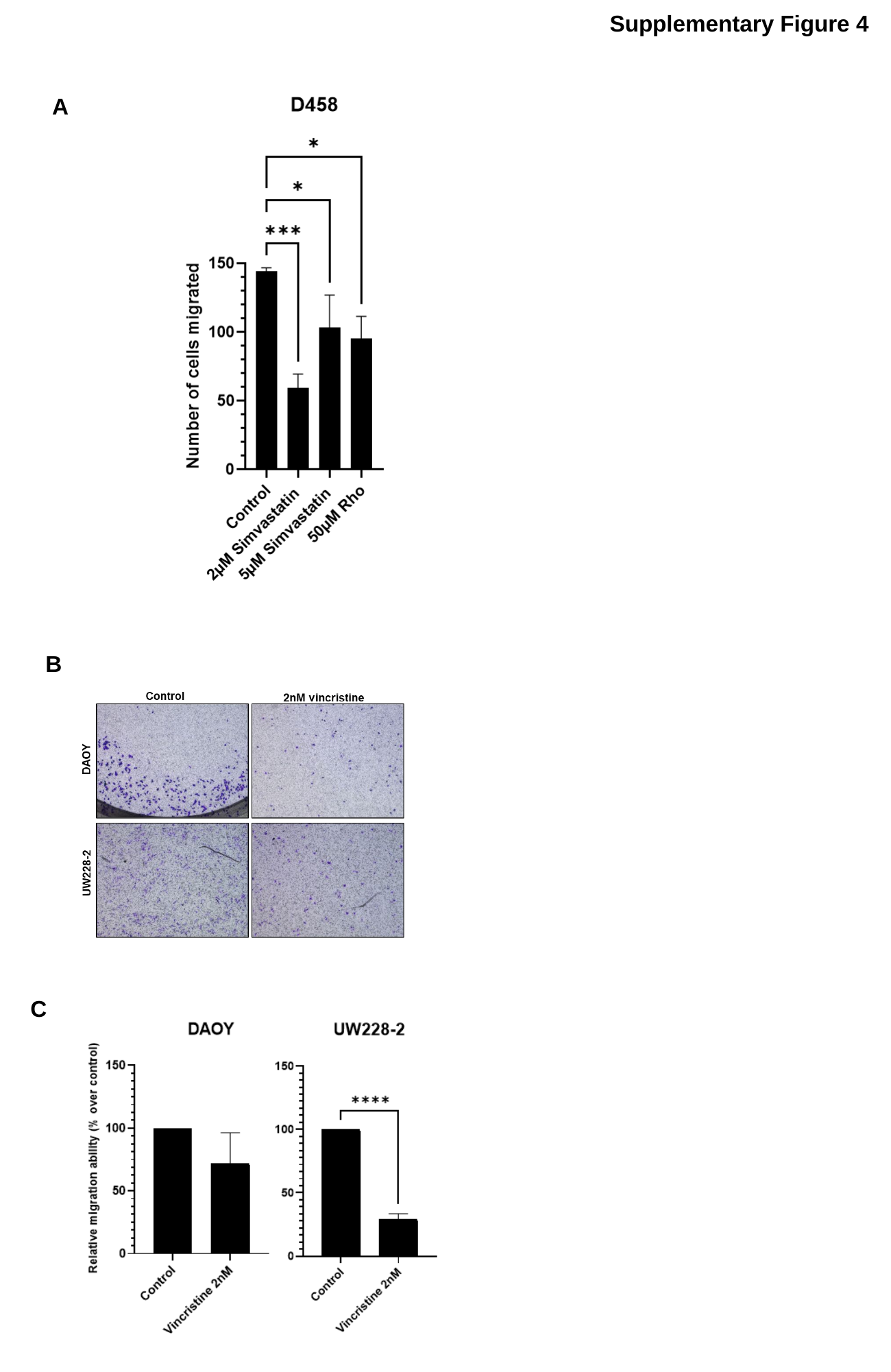

Supplementary Figure 4
A
B
C

## Slide 5
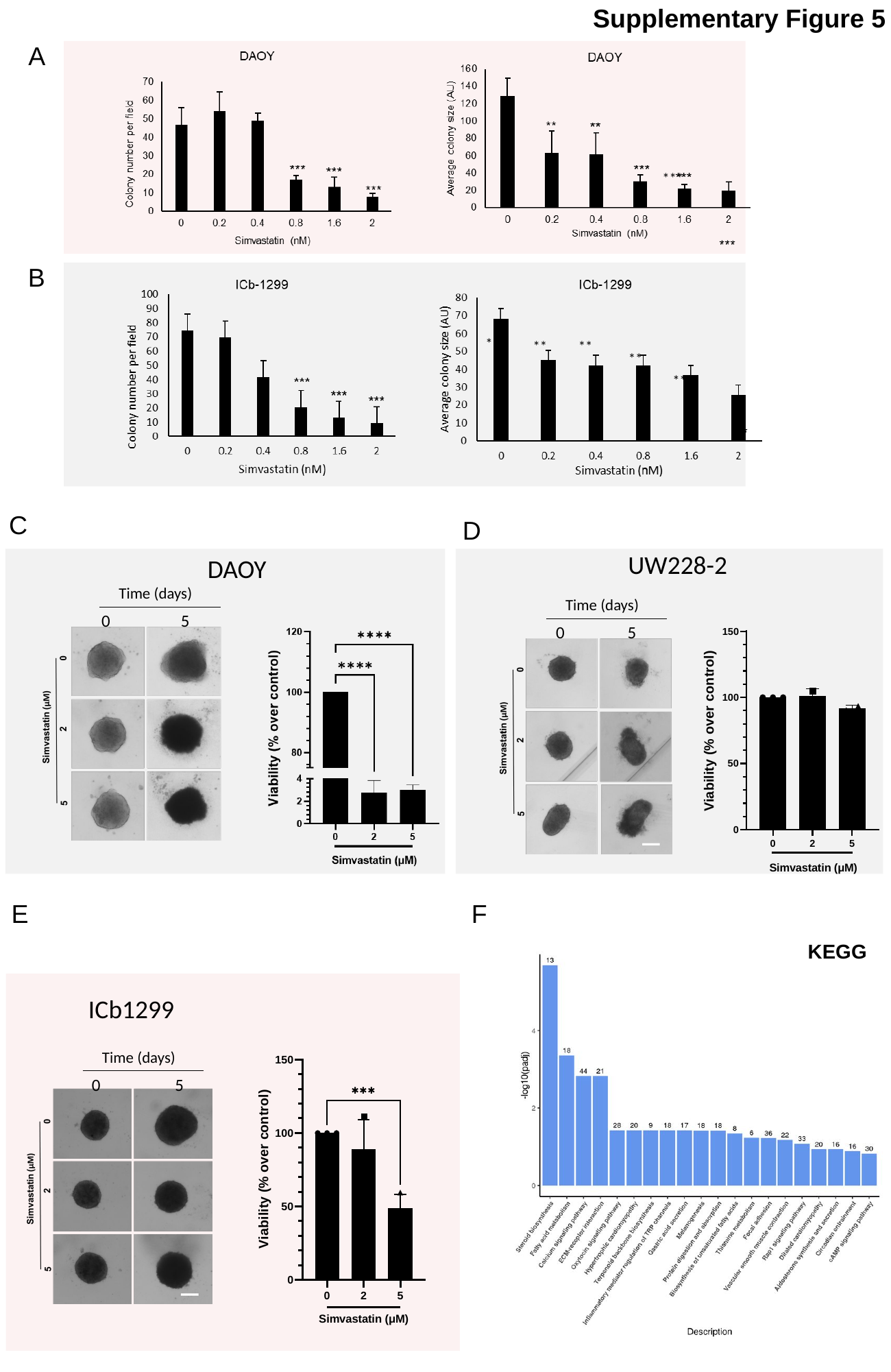

Supplementary Figure 5
A
***
B
**
**
**
**
***
C
D
UW228-2
DAOY
Time (days)
0 5
Time (days)
 0 5
E
F
KEGG
ICb1299
Time (days)
0 5

## Slide 6
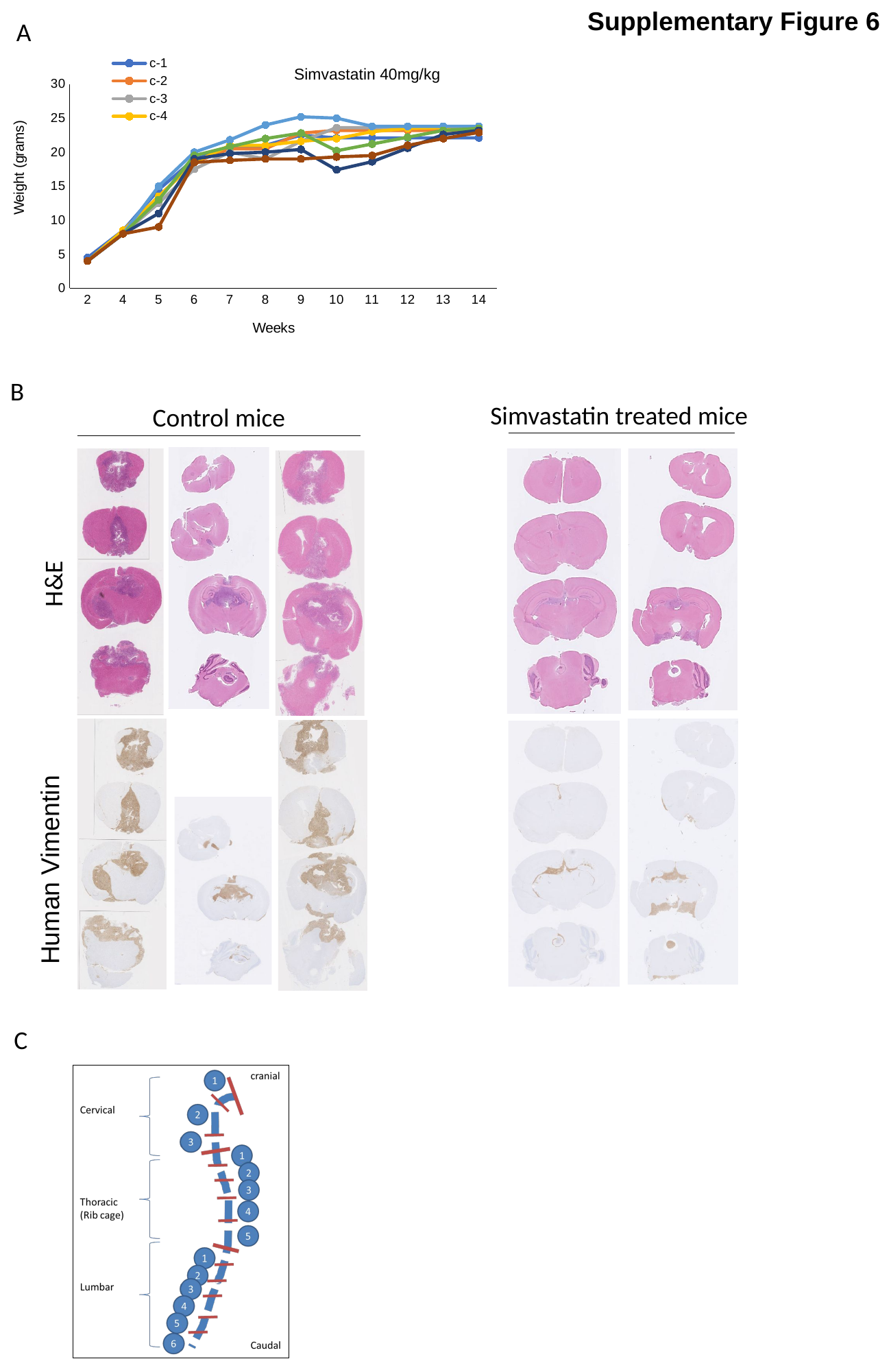

Supplementary Figure 6
A
### Chart
| Category | c-1 | c-2 | c-3 | c-4 | s-1 | s-2 | s-3 | s-4 |
|---|---|---|---|---|---|---|---|---|
| 2 | 4.5 | 4.0 | 4.0 | 4.0 | 4.0 | 4.0 | 4.0 | 4.0 |
| 4 | 8.5 | 8.0 | 8.0 | 8.5 | 8.0 | 8.0 | 8.0 | 8.0 |
| 5 | 14.5 | 13.5 | 12.5 | 13.5 | 15.0 | 13.0 | 11.0 | 9.0 |
| 6 | 19.0 | 18.5 | 17.5 | 18.5 | 20.0 | 19.5 | 19.0 | 18.5 |
| 7 | 20.5 | 20.5 | 20.0 | 21.0 | 21.8 | 20.8 | 19.8 | 18.8 |
| 8 | 21.0 | 20.5 | 19.0 | 21.0 | 24.0 | 22.0 | 20.0 | 19.0 |
| 9 | 22.5 | 22.8 | 21.6 | 21.6 | 25.2 | 22.8 | 20.4 | 19.0 |
| 10 | 22.1 | 23.2 | 23.6 | 22.0 | 25.0 | 20.2 | 17.4 | 19.3 |
| 11 | 22.1 | 23.2 | 23.6 | 23.0 | 23.8 | 21.2 | 18.6 | 19.5 |
| 12 | 22.1 | 23.2 | 23.6 | 23.5 | 23.8 | 22.2 | 20.6 | 21.0 |
| 13 | 22.1 | 23.2 | 23.6 | 23.6 | 23.8 | 23.2 | 22.6 | 22.0 |
| 14 | 22.1 | 23.2 | 23.6 | 23.6 | 23.8 | 23.5 | 23.2 | 22.9 |Simvastatin 40mg/kg
B
Simvastatin treated mice
Control mice
H&E
Human Vimentin
C

## Slide 7
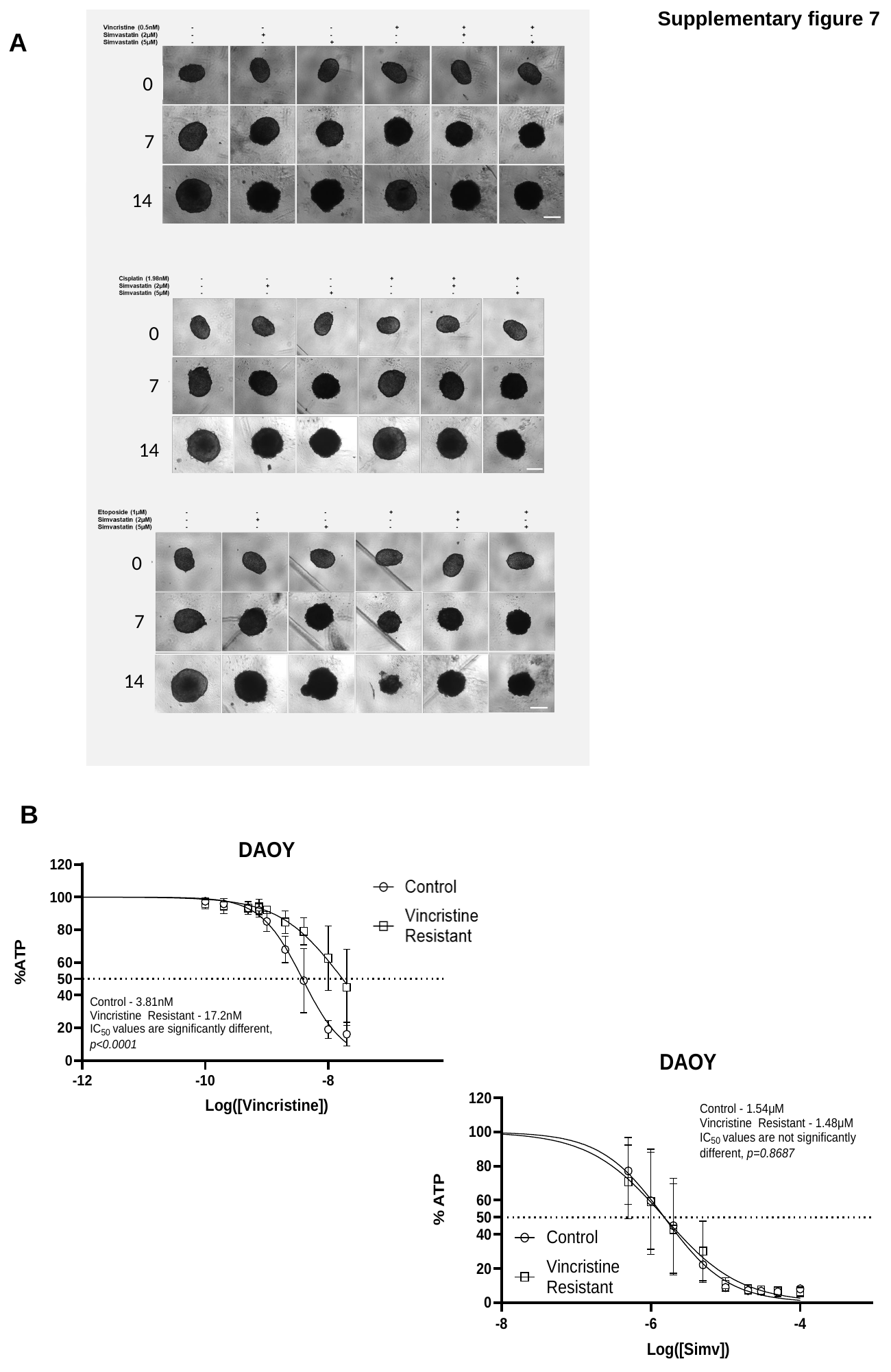

Supplementary figure 7
A
0
7
14
0
7
14
0
7
14
B

## Slide 8
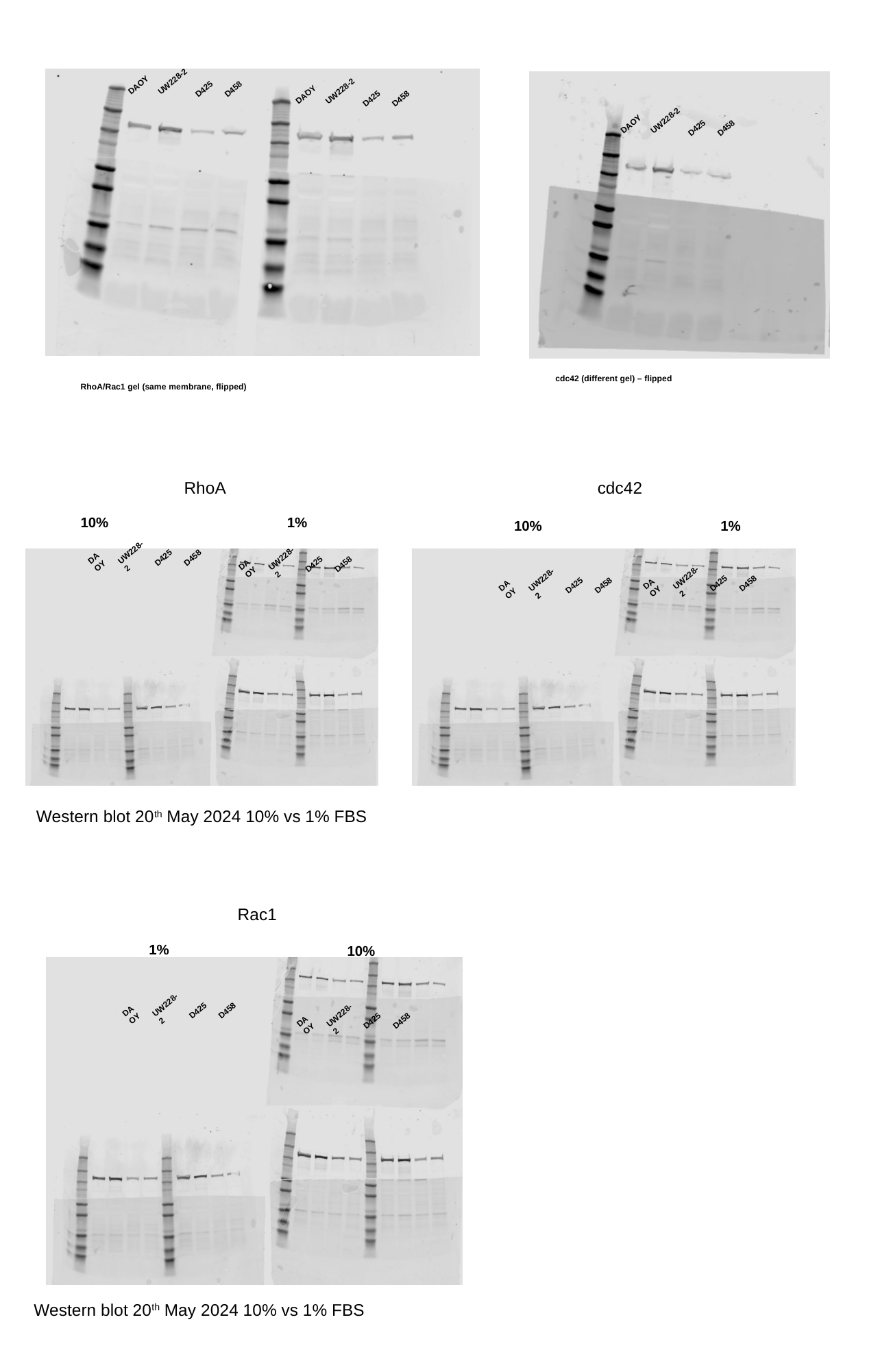

UW228-2
UW228-2
DAOY
D425
D458
DAOY
D425
D458
UW228-2
DAOY
D425
D458
cdc42 (different gel) – flipped
RhoA/Rac1 gel (same membrane, flipped)
RhoA
cdc42
10%
1%
10%
1%
UW228-2
DAOY
UW228-2
D425
D458
DAOY
D425
D458
UW228-2
UW228-2
DAOY
DAOY
D425
D458
D425
D458
Western blot 20th May 2024 10% vs 1% FBS
Rac1
1%
10%
UW228-2
DAOY
D425
D458
UW228-2
DAOY
D425
D458
Western blot 20th May 2024 10% vs 1% FBS

## Slide 9
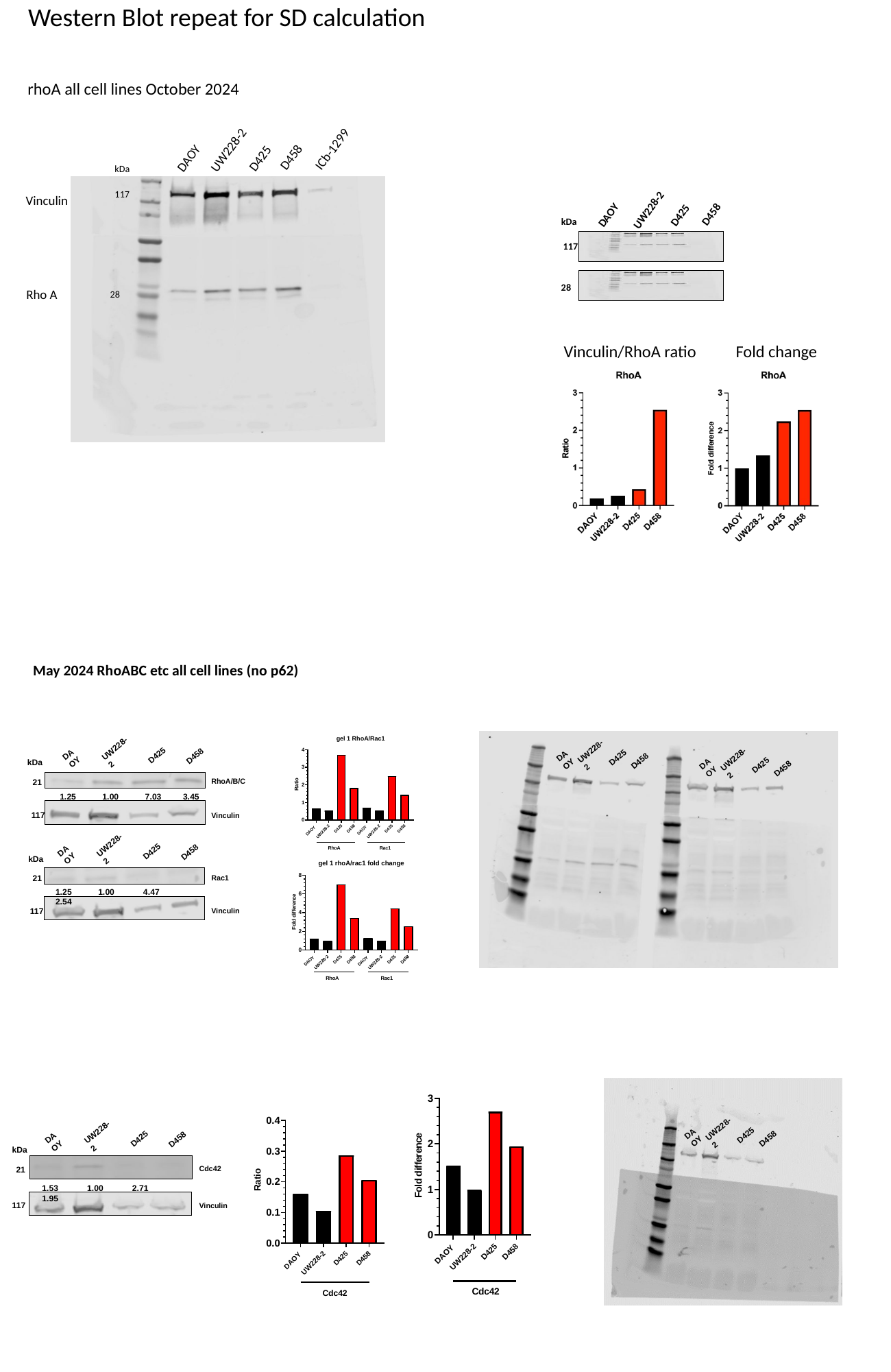

Western Blot repeat for SD calculation
rhoA all cell lines October 2024
ICb-1299
UW228-2
D458
D425
DAOY
kDa
117
Vinculin
UW228-2
D458
D425
DAOY
kDa
117
28
Rho A
28
Vinculin/RhoA ratio
Fold change
May 2024 RhoABC etc all cell lines (no p62)
UW228-2
UW228-2
DAOY
DAOY
D425
D458
UW228-2
D425
DAOY
D458
kDa
D425
D458
21
RhoA/B/C
1.25 1.00 7.03 3.45
117
Vinculin
UW228-2
DAOY
D425
D458
kDa
21
Rac1
1.25 1.00 4.47 2.54
Vinculin
117
UW228-2
UW228-2
DAOY
D425
DAOY
D458
D425
D458
kDa
21
Cdc42
1.53 1.00 2.71 1.95
117
Vinculin

## Slide 10
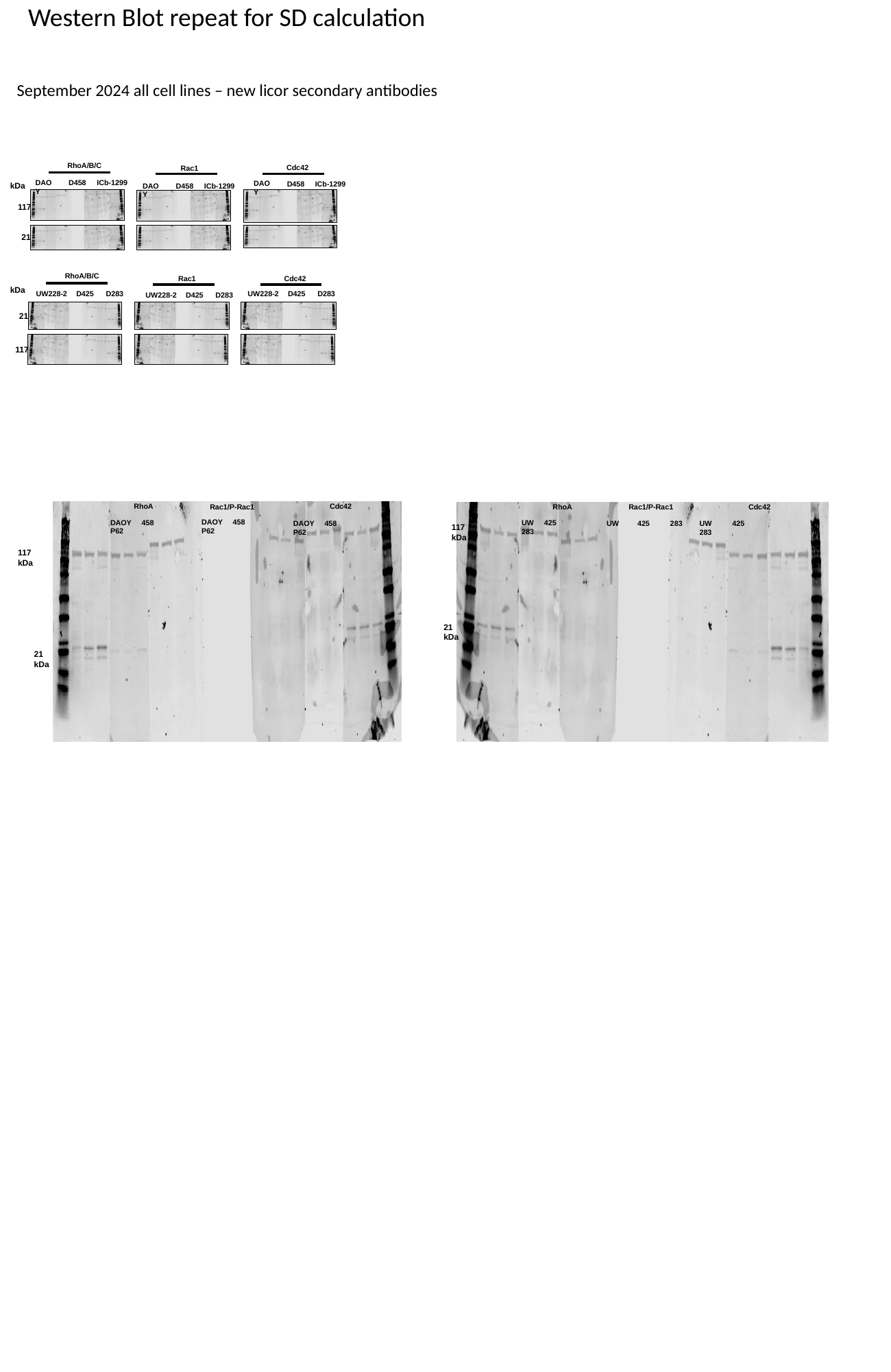

Western Blot repeat for SD calculation
September 2024 all cell lines – new licor secondary antibodies
RhoA/B/C
Cdc42
Rac1
DAOY
D458
ICb-1299
DAOY
D458
ICb-1299
kDa
DAOY
D458
ICb-1299
117
21
RhoA/B/C
Cdc42
Rac1
kDa
UW228-2
D425
D283
UW228-2
D425
D283
UW228-2
D425
D283
21
117
Cdc42
RhoA
Rac1/P-Rac1
DAOY 458 P62
DAOY 458 P62
DAOY 458 P62
117 kDa
21 kDa
Cdc42
RhoA
Rac1/P-Rac1
UW 425 283
UW 425 283
UW 425 283
117 kDa
21 kDa

## Slide 11
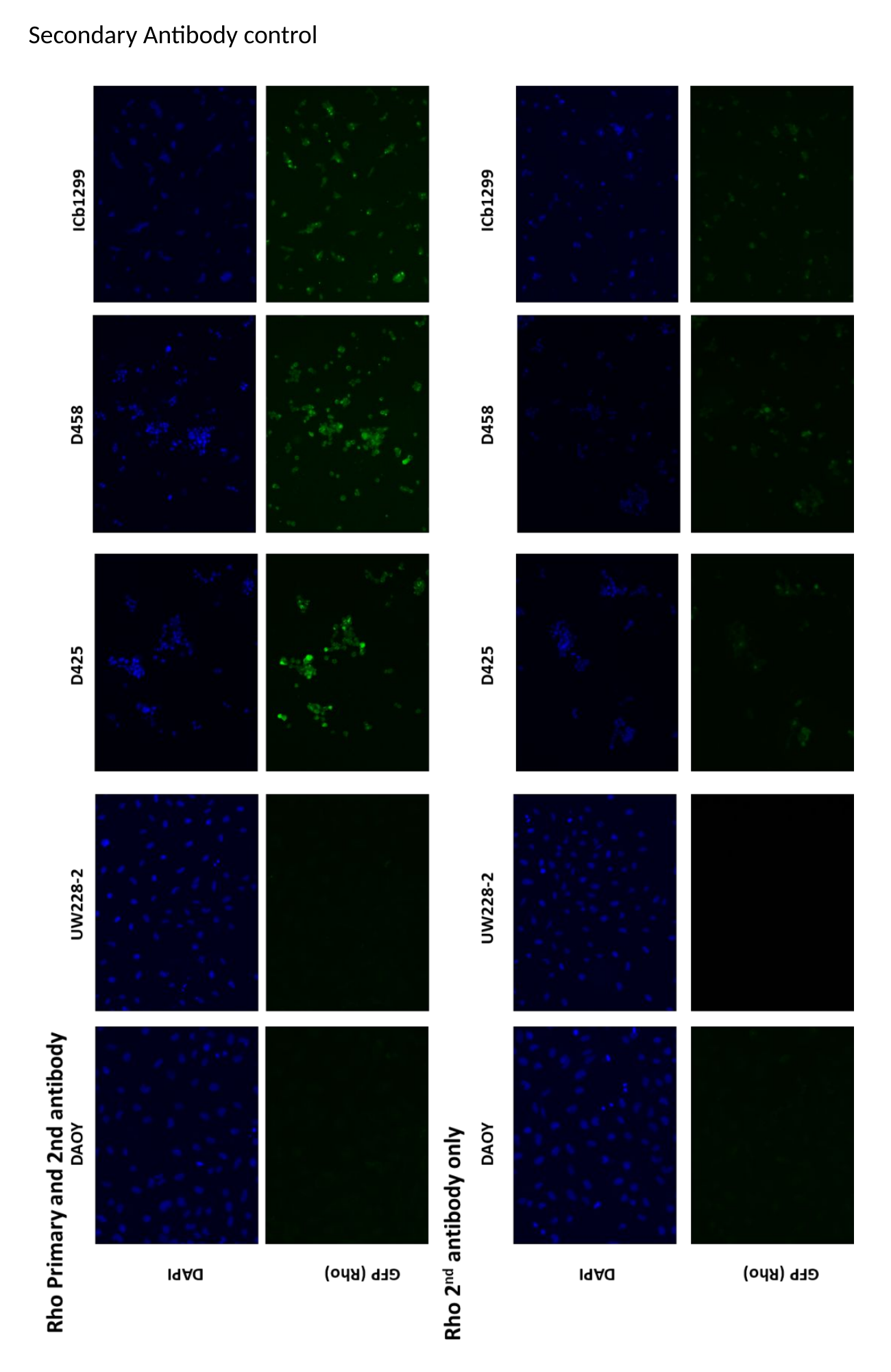

Secondary Antibody control

## Slide 12
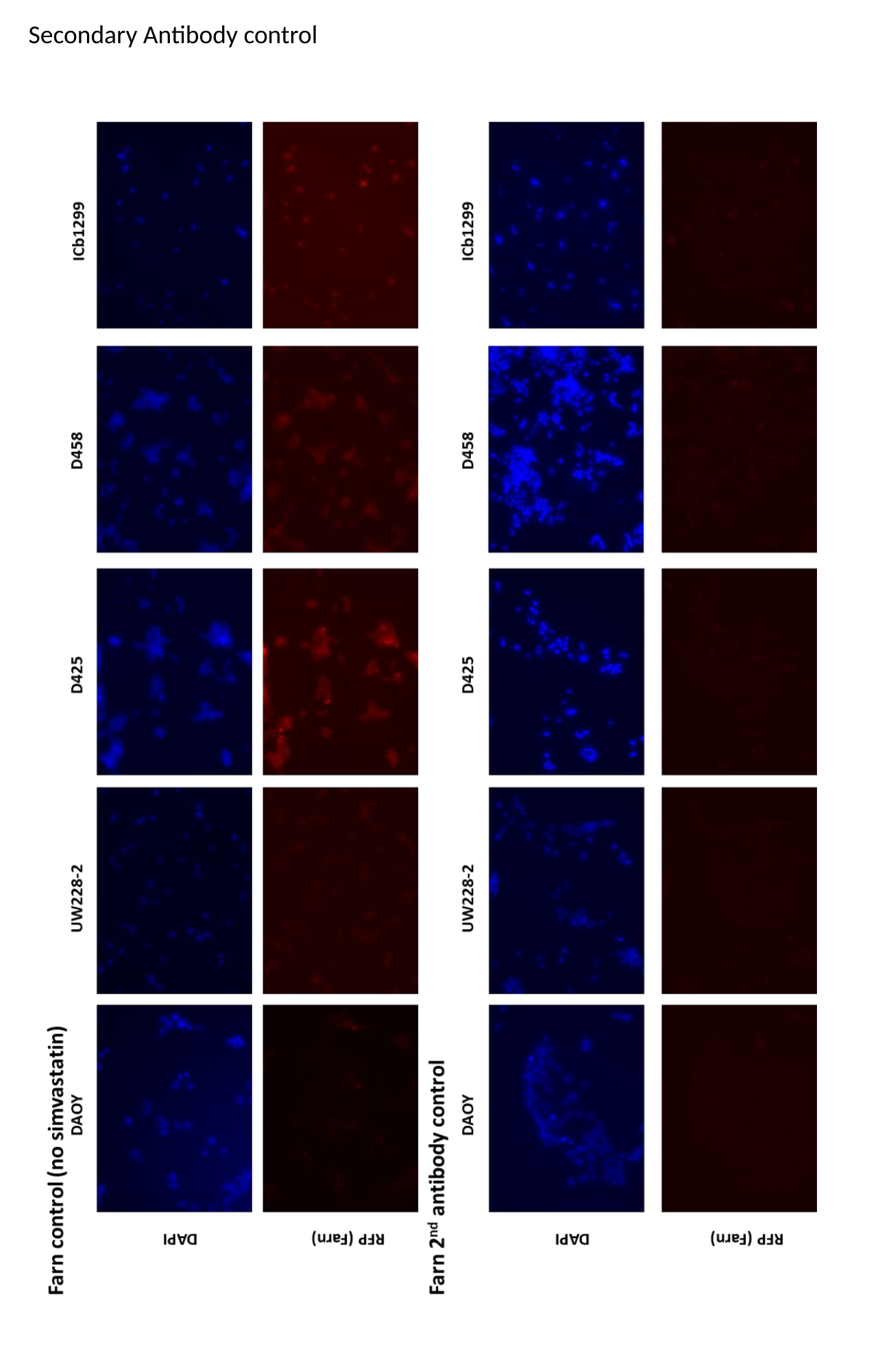

Secondary Antibody control
