# Supplementary material for: Simvastatin suppresses spinal cord metastasis of medulloblastoma at clinically significant doses
Source: Cell Death Dis. 2025 Jul 15;16(1):527. doi: 10.1038/s41419-025-07829-0 (PMC12263873; doi:10.1038/s41419-025-07829-0)
Supplement: Supplementary file 2 — Supplementary Figure legends [file 41419_2025_7829_MOESM2_ESM.docx]

**Supplementary Material:**

**Supplementary Figure Legends:**

**Supplementary Figure 1. The mevalonate pathway is upregulated in SHH-MBs and G3/G4-MBs.** A) Correlation analysis of *HMGCR* versus *SREBF2* genes shows a positive correlation (R:0.362) between SREBF2 expression and HMGCR expression in MBs. P<0.0001. B) Medulloblastoma (Pfister dataset; n=223), ependymoma (Pfister dataset; n=209), Glioma (Paugh dataset; n=53), DIPG (Paugh dataset; n=23) and normal cerebellum (Roth dataset; n=9) datasets were used to compare gene expression between paediatric brain tumours and normal tissue. Expression data is displayed as box and whisker plots that shows the upper quartile, median, and lower quartile range as well as the sample maximum and minimum as indicated by the error bars. Significant differences in HMGCR expression between groups were calculated using a Brown-Forsythe and Welch ANOVA test with Dunnett’s T3 multiple comparisons post-hoc test; **p ≤ 0.01, ***p ≤ 0.001. C) Expression of *HMGCR, IDI1* and *FDPS* genes in different MBs RNA sequencing datasets compared with normal cerebellum data. All bioinformatic analysis was done using the R2 genomics platform using a normal brain dataset (172 cases) and Gilbertson (76), Pfister (73), Hsieh (31), denBoer (51), Pfister (223), Delattre (57) and Kool (62). D) Kaplan–Meier survival curves based on high and low HMGCR expression levels in WNT and SHH-MBs tumours derived from Cavalli cohort with 763 patients (P=0.11; P=0.07). E) Mass spectrometry standard with cholesterol. Graph shows the residual and response of different doses of cholesterol (250, 500, 1000, 2000, 4000 and 8000µM). F) Quantification of mean fluorescent of soluble cholesterol from 3 independent experiments in UW228-2, D425 and D458 cells performed with GraphPad Prism. One-way ANOVA performed with Tukey’s post-Hoc comparisons, where ** = P<0.01. Amplex™ Red Cholesterol Assay Kit was used to measure free cholesterol.

**Supplementary Figure 2. Rho-GTPase signalling supports in SHH-MBs and G3/G4-MBs cell migration.** A) Diagram of the mevalonate pathway with key enzymes and function of the intermediates. B) Levels of Cdc42 and Rac1 in DAOY, UW228-2, D425 and D458 cells, determined via western blotting. Fold change in protein levels compared with lowest expressing sample. C) Representative western blotting of RhoA, Rac1 and Cdc42 levels in DAOY, UW228-2, D425 and D458 in serum starved conditions (1%). D) Fold differences for RhoA, Rac1 and Cdc42 displayed numerically and graphically in serum starved conditions (1%). E) Quantification of mean fluorescence intensities from 3 independent experiments of anti-RhoA/B/C in DAOY, UW228-2, D425, D458 and ICb-1299 cells after a single treatment with 5uM simvastatin for 6 and 16 hours.

**Supplementary Figure 3. Simvastatin induces significant transcriptomic changes in MBs cells.** A-B) DAOY, D425 and ICb-1299 cells were treated with simvastatin for 72h before the cells were harvested for RNA-seq library preparation. A) KEGG and (B) GO enrichment analyses (p < 0.05 and q < 0.05). C) MTT assay in DAOY, UW228-2 and D458 after 2 and 5 uM simvastatin after 48 h treatment. D) Representative scatter plots of Annexin V/PI staining to assess the apoptotic ratios of DAOY control or simvastatin (5 uM) treated for 48 h. Percentage of viable cells in control, 5 uM simvastatin. All data were collected from three independent experiments and are presented as the mean ±SD. E) Cell-cycle analysis with PI staining of DAOY, UW228-2, D425, D458 and ICb-1299 MBs cells in the presence of 10µM simvastatin at 0, 16, 22 and 28h. Data gathered and analysed using FACS Canto and FCS Express 7 software.

**Supplementary figure 4. Simvastatin reduce SHH-MBs and G3/G4-MBs cell migration.** A) Transwell migration analysis of D458 cells in the presence of simvastatin (2 and 5uM) and the ROCK inhibitor Y-27632 (50uM). B) Representative images of a transwell migration analysis of DAOY and UW29-2 cells in the presence of Vincristine (2nM). C) Quantification of migrated DAOY and UW228-2 cells in the presence of vincristine carried out using ImageJ software. One-way ANOVA statistical analysis done using GraphPad Prism software. Wound closure assay of DAOY and UW228-2 cells over a period of 21h.

**Supplementary figure 5. Simvastatin reduced SHH-MBs and G3/G4-MBs colony formation in soft agar.** A) Quantification of colony number and colony size in soft agar assay in DAOY cells. B) Quantification of colony number and colony size in soft agar assay in ICb-1299 cells. C-E) Representative micrographs of DAOY, UW228-2 and ICb1299 spheroids after 5 days treatment with 2 and 5µM simvastatin treatment. Cell viability was measured with CellTiter Glo and quantification has been shown. *** = P<0.001 and **** = P<0.0001. F) DAOY, D425 and ICb-1299 cells were culture in 2D and 3D conditions before the cells were harvested for RNA-seq library preparation. KEGG enrichment analyses (p < 0.05 and q < 0.05).

**Supplementary figure 6. Low doses of simvastatin result in significantly improved survival in an orthotopic MBs xenograft model.** A) Mouse weight over 14 weeks showing that simvastatin was well tolerated. B) Coronal sections of the entire brain of control and 40 mg/Kg simvastatin treated mice. Sections were stained with H&E and human vimentin. C) Diagram of the coronal sections of the entire spinal cord, cervical, thoracic and lumbar of xenograft mouse which were examined for cytology.

**Supplementary Figure 7. Effect of simvastatin plus cisplatin drug and on resistant SHH-MBs and G3/G4-MBs cells.** A) Morphology of UW228-2 spheroids after 7- day and 14-day treatment with simvastatin (2 and 5µM), with or without 0.5nM vincristine, 1.98 nM cisplatin or 1uM etoposide. One-way ANOVA performed with Tukey’s post-hoc comparisons: (*) P < 0.05; (***) P < 0.001. B) To obtain the IC50, DAOY control (ᵒ) cells and vincristine resistant cells (⸋) were exposed to growing concentrations of vincristine and simvastatin for 24 h. ATP levels were measured with CellTiter-Glo® Luminescent Cell Viability Assay (Promega, US).
